# Scale-dependent foraging behaviour and habitat associations of two sympatric marine top predators

**DOI:** 10.1007/s10980-025-02281-z

**Published:** 2025-12-27

**Authors:** Matt I. D. Carter, Geert Aarts, Sophie M. J. M. Brasseur, Gordon D. Hastie, Simon E. W. Moss, Jacob Nabe-Nielsen, Jonas Teilmann, Dave Thompson, Paul M. Thompson, Cécile Vincent, Debbie J. F. Russell

**Affiliations:** 1 Sea Mammal Research Unit, School of Biology, University of St Andrews, St Andrews, UK; 2 Wageningen Marine Research, Wageningen University & Research, Den Helder, The Netherlands; 3 Royal Netherlands Institute for Sea Research, Texel, The Netherlands; 4 Section for Marine Mammal Research, Department of Ecoscience, Aarhus University, Roskilde, Denmark; 5 Lighthouse Field Station, School of Biological Sciences, University of Aberdeen, Cromarty, UK; 6 Pelagis, UAR 3462, University of La Rochelle/CNRS, La Rochelle, France; 7 Centre for Research into Ecological & Environmental Modelling, University of St Andrews, St Andrews, UK

**Keywords:** Area-restricted search (ARS), Central-place forager, Habitat association model, Hidden Markov model (HMM), Pinnipeds, Seascape ecology

## Abstract

**Context:**

Theoretical research has considered how animals should optimise foraging strategies to maximise fitness, adapting search scale to exploit different habitats and minimise competition. Empirical studies have described multi-scale area-restricted search (ARS) strategies for some species, but the physical and biological mechanisms underpinning such behaviour are rarely studied.

**Objectives:**

Our objectives were to quantify the presence, prevalence, and habitat associations of scale-dependent foraging for two sympatric seal species, accounting for regional variation across the seascape.

**Methods:**

We analyse a GPS telemetry dataset of 116 grey (*Halichoerus grypus*) and 325 harbour seals (*Phoca vitulina*) tracked throughout the North Sea. We test the existence of multi-scale ARS, comparing hidden Markov models (HMMs) with two ARS states against more conventional HMMs (one ARS state). We quantify regional variation and examine the scale-dependence of foraging habitat associations using *post-hoc* “use-encounter” models.

**Results:**

Both species exhibited nested broad-scale and focussed ARS. Accounting for scale resulted in increases of up to 25% and 46% in inferred ARS for grey and harbour seals respectively. The prevalence and habitat associations of different ARS scales varied in a regional species-specific manner.

**Conclusions:**

We demonstrate the first application of HMMs to capture multi-scale ARS from animal-borne tracking data. Overlooking scale-dependence may mask individual variation and underestimate ARS, with consequences for ecological understanding and conservation applications. We hypothesise that seals employ different search scales for different habitats, competition levels and/or prey types. We call for further research to elucidate the prevalence and ecological significance of this phenomenon in other aquatic predators.

**Supplementary Information:**

The online version contains supplementary material available at 10.1007/s10980-025-02281-z.

## Background

The need to acquire foraging resources shapes the behaviour, distribution, and life history of all animals (Charnov 1976; Stephens and Krebs 1986). Understanding how mobile animals optimise movement behaviours to search for, find, and exploit prey while balancing energetic expenditure and gain and avoiding competition and predation is a key question in ecology (Pyke et al. 1977). Optimal Foraging Theory suggests that animals should adapt the scale of their search patterns in response to changes in habitat composition and prey density to maximise foraging dividends (Ritchie 1998). However, a mechanistic understanding of foraging decision-making in free-ranging animals is lacking for many species (McGarigal et al. 2016). Unpicking the physical and biological processes that shape foraging behaviour can elucidate the drivers of species distributions and facilitate assessment of population responses to anthropogenic habitat modification (Tucker et al. 2018; Cooke et al. 2024) and climate change (Robinson et al. 2009; Hindell et al. 2017; Muhling et al. 2025). Such knowledge is also essential to understanding the structure and functioning of ecosystems, and to mitigating the biodiversity crisis (Singh 2002).

For wide-ranging marine predators such as marine mammals, seabirds and pelagic fish, the challenge of finding patchily distributed prey resources is further complicated by the dynamic, fractal nature of the environment (Russell et al. 1992; Pirotta et al. 2014). Individuals frequently cover large distances while foraging, encountering a range of different environmental conditions and habitat types. Animal-borne biologging devices allow tracking of movement, alongside collection of physiological and environmental data which can either be remotely transmitted or recovered, providing invaluable insight into the lives of free-ranging animals that would otherwise be difficult to monitor (Cooke et al. 2004; Ropert-Coudert et al. 2009). Tracking central-place foragers such as pinnipeds and breeding seabirds provides opportunities to link at-sea behaviour and distribution to population trends monitored at coastal breeding and haulout sites (Hindell et al. 2017). Moreover, they must navigate between the central place and offshore foraging grounds, leading to a natural categorisation of at-sea behaviours into directed transit (i.e., moving between the central place and foraging grounds, or between foraging grounds) and area-restricted search (ARS) (i.e., foraging) (Dorfman et al. 2022).

Over the past two decades, analytical options have been developed to decode biologging data into inference of behaviour, each with their own merits and limitations (Fauchald and Tveraa 2003; Patterson et al. 2008; Carter et al. 2016). Many approaches use track geometry to distinguish encamped movements with short step lengths and high turn angles (inferred as ARS) from directed movements with long step lengths and low turn angles (inferred as transit or migration) (Dorfman et al. 2022). Before the popularisation of more sophisticated techniques, such as state-space models (SSMs), ARS was commonly inferred from first-passage time (FPT), where time to cross a virtual circle exceeds a given threshold (Fauchald and Tveraa 2003). Determining the appropriate circle radius requires trialling a range of values, and FPT studies may therefore implicitly assume that ARS occurs at multiple spatial scales. Many seabird species display ARS at nested scales, adapting search patterns in response to prey encounters (Pinaud and Weimerskirch 2005, 2007; Weimerskirch et al. 2007; Pinaud 2008; Hamer et al. 2009). For example, FPT analysis showed that breeding northern gannets (*Morus bassanus*) exhibited hierarchical search strategies, with fine-scale ARS nested within broader search patterns (Hamer et al. 2009). Estimates of foraging effort inferred from diving behaviour suggested that birds had higher prey encounter rates when using nested-scale than single-scale ARS strategies (Hamer et al. 2009). Multi-scale ARS is comparatively under-studied in pinnipeds; Thums et al. (2011) identified multiple scales of ARS in individual northern elephant seals (*Mirounga leonina*) using FPT, although this was secondary to the focus of the study and scale nesting was not examined.

In recent years, SSMs, in particular hidden Markov models (HMMs), have emerged as a powerful and flexible option for animal movement data, with user-friendly software packages facilitating complex ecological analyses (Michelot et al. 2016; McClintock and Michelot 2018; Jonsen et al. 2023). HMMs assume that observed movement patterns arise from a finite number of discrete unobserved behavioural states (Zucchini et al. 2016). HMMs can incorporate multiple data streams to disentangle behaviours that may otherwise be conflated. For example, by including dive metrics alongside track geometry, McClintock et al. (2013) demonstrated that harbour seals (*Phoca vitulina*) frequently spend prolonged periods at sea on the surface. Not accounting for such behaviour could overestimate foraging activity and bias activity budget estimates. Moreover, ground-truthing studies have found that HMMs frequently outperform alternative methods, such as FPT, at correctly identifying foraging behaviour (Dragon et al. 2012; Bennison et al. 2018). HMMs have become the “gold standard” analytical approach for tracking data from diverse taxa, including pinnipeds (Carter et al. 2016) and seabirds (Akeresola et al. 2024). However, unlike FPT analysis, where the scale at which ARS is detected is inherently flexible, because the number of states is pre-defined by the user in an HMM, previous studies have fitted only one ARS state which is assumed to capture all foraging behaviour (Carter et al. 2016), thus precluding investigation of multi-scale foraging strategies.

A further feature of HMMs is the ability to link behavioural inference to the environment. The influence of covariates on the probability of switching between states can be modelled within the HMM, allowing environmental conditions to influence state assignment (Morales et al. 2004). Studies have used this approach to investigate species-habitat associations for marine predators (Patterson et al. 2009; Grecian et al. 2018; Wyles et al. 2022). For example, Wyles et al. (2022) found that grey seals (*Halichoerus grypus*) in the North Sea were more likely to switch from transit into ARS upon encountering slope-like features on the seabed. Although this approach offers insights into the mechanisms underpinning behavioural switches, it does not necessarily reveal the environmental characteristics of foraging areas (Glennie et al. 2023). For example, animals may switch into ARS upon encountering cues indicating proximity to foraging habitat, but the habitat where foraging activity is concentrated may be different. Alternatively, HMMs can be integrated with step selection functions (SSFs; where the observed location of an animal is compared to randomly sampled locations representing possible movement choices at each time step) to understand behaviour-specific habitat selection in the context of what habitat is available (Nicosia et al. 2017; Klappstein et al. 2023). A third option is to use state estimates from the HMM as response terms in *post-hoc* logistic regression models (Kane et al. 2020; Stalder et al. 2020; Nykänen et al. 2025). This two-step “use-encounter” approach quantifies the probability that an animal will forage in a habitat given that it is encountered, thus explicitly considering the environment associated with ARS. Such models can therefore provide insight on both the mechanisms of foraging habitat selection and the characteristics of foraging areas. Nevertheless, regardless of method, the vast majority of species-habitat modelling studies do not consider scale-dependent habitat selection, and much less behaviour-specific habitat associations at multiple spatial scales (McGarigal et al. 2016).

Grey and harbour seals have been model species for the development of state-space techniques for inferring behaviour from animal-borne tracking data (Jonsen et al. 2005; McClintock et al. 2013; van Beest et al. 2019; Carter et al. 2020) and their at-sea behaviour has been examined in detail since the first tracking studies over thirty years ago (Thompson and Miller 1990; Thompson et al. 1991; McConnell et al. 1992). Much of this research has focussed on the North Sea, which is an important population centre for both species (Banga et al. 2022). They are sympatric throughout the North Sea, exhibiting species-specific regional differences in diet (Wilson and Hammond 2019), behaviour (Russell et al. 2015), distribution (Carter et al. 2025), and population trends (Brasseur et al. 2018; Russell et al. 2019; Thompson et al. 2019). However, to date the existence of multi-scale ARS remains untested. Some HMM studies have fitted models for these species at the individual level (no pooling) due to individual differences in movement characteristics (Russell et al. 2015; Wyles et al. 2022), which may point to differences in search scale. However, individual-level models hinder our ability to draw population-level conclusions about the characteristics of behavioural states (Glennie et al. 2023). By contrast, population-level HMMs (complete pooling) allow such conclusions, but risk mis-specifying behaviour in some individuals if the number of possible states does not accommodate individual variation in characteristics such as search scale (Glennie et al. 2023). Population-level HMMs with multiple ARS states may represent a solution.

The aim of this study is to examine the prevalence and habitat associations of scale-dependent foraging for grey and harbour seals across the North Sea. We model a GPS satellite telemetry dataset, exceptional among marine vertebrate studies in both sample size (116 grey and 325 harbour seals) and spatial coverage (encapsulating most centres of abundance in the North Sea). Specifically, we address three objectives for both species: (i) characterise foraging behaviour, testing the presence of single vs multi-scale ARS, using population-level HMMs; (ii) quantify regional differences in the prevalence of multi-scale ARS; and (iii) quantify regional differences in the scale-dependence of foraging habitat associations using *post-hoc* models. We discuss our findings in the context of current foraging theory, animal movement analysis and seascape ecology, and highlight priorities for future work.

## Methods

### Instrumentation

**Fig. 1:**
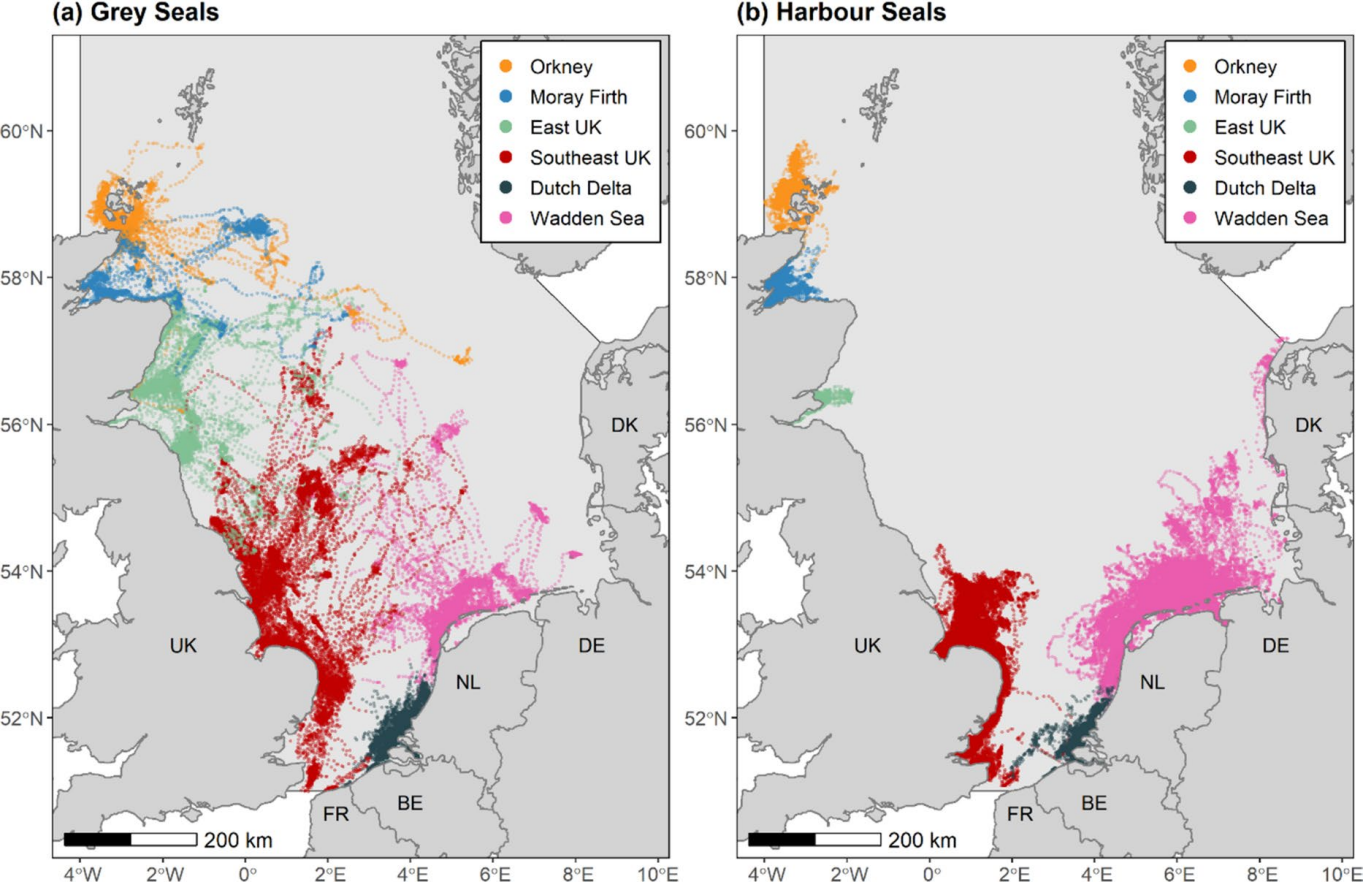
Tracking data from **a** 116 grey and **b** 325 harbour seals with trips attributed to six regions around the North Sea based on haulout locations, denoted by different colours. Light grey polygon denotes the North Sea study area (ICES Area 4). UK = United Kingdom, FR = France, BE = Belgium, NL = Netherlands, DE = Germany, DK = Denmark

Animal-borne satellite telemetry tags (Fastloc® GPS-GSM; SMRU Instrumentation, United Kingdom (UK)) were deployed on grey and harbour seals along the coast of the UK, France, and the Netherlands between 2007 and 2019 (Fig. 1). Seals were caught at haulouts using either seine, pop-up, tangle or hand nets and a tag was glued to the fur on the neck, falling off by the end of the annual moult. Data were pooled across years for analyses to maximise spatial coverage and ensure adequate sample size. The final sample size for habitat association analysis was reduced through data cleaning protocols and geographic restriction of trips to the North Sea study area to 116 grey (F:61, M:54, Unknown:1) and 325 (F:170, M:160) harbour seals from an available dataset of 149 grey and 401 harbour seals (see “Foraging Habitat Association Models” below). All seals were deemed to be at least six months old, and thus likely to be exhibiting adult-like at-sea behaviour (Carter et al. 2020).

### Telemetry data processing

Tags collected and transmitted locational, dive and haul-out records, as well as summaries of proportion of time spent diving or hauled-out in regular 2 or 4 h intervals depending on tag parametrisation. Location data were first cleaned to remove erroneous location estimates using a minimum threshold of five satellite links, and a maximum residual error value of 25 (Russell et al. 2015). Since seals make discrete foraging trips at sea punctuated by haul-out events on land, a unique identifier was assigned for each trip, defined as any at-sea location data between recorded haul-out events. Erroneous haul-out events (when the seal is at sea with the wet/dry sensor exposed at the surface and sufficiently dry for > 10 min) were excluded following the protocol outlined in Russell and Carter (2021).

Cleaned location data were regularised to a constant 2 h time step using linear interpolation, synchronised to the midpoint of 2 h summary data intervals. Where summary data were recorded in 4 h intervals (n = 128 tags), 2 h summary data were created based on the detailed dive and haulout records and quality checked to ensure comparability with the rest of the dataset. Following Carter et al. (2020), a regularised location was flagged as “unreliable” if there were missing summary data, or a gap > 6 h between the surrounding observed location fixes. Any tag that had persistent “unreliable” intervals leaving no identifiable foraging trips (15 grey and 75 harbour seals) was removed from the dataset. All data processing, analysis, and figure creation was carried out in R (R Core Team 2023).

### Hidden markov models

To characterise the behaviour of seals, multivariate discrete-time HMMs were fitted to the cleaned regularised location data for each species using the R package “momentuHMM” (McClintock and Michelot 2018). To address Objective 1 and determine if a model capturing two scales of ARS was more appropriate than the conventional approach with one ARS state (sensu Jonsen et al. (2005); Russell et al. (2015); Carter et al. (2020); Iorio-Merlo et al. (2022)) at the population level, models were fitted per species with (i) four states: “ARS”, “transit”, “non-diving”, and “unknown”, and (ii) five states: “focussed ARS”, “broad ARS”, “transit”, “non-diving”, and “unknown”. The “non-diving” state captures both haulout and at-sea surface activity, while the “unknown” state captures intervals where location and/or summary data were missing (see below). Bayesian Information Criterion (BIC) scores were then used to compare these two models and determine the most appropriate number of states. BIC is a more conservative alternative to Akaike Information Criterion (AIC) for HMM order selection, but since even BIC tends towards more complex models, such metrics should be used only as a guideline (Pohle et al. 2017). Thus, final selection was made following the “pragmatic order selection” protocol outlined in Pohle et al. (2017), including close scrutiny of state-dependent distributions and mapped state assignments for biological plausibility.

The “non-diving” state was coded as a “known state” based on the time spent diving from tag summary records. Following Russell et al. (2015), for each 2 h time interval (*t*), the state $(Z_{t} )$ was set a priori as “non-diving” $(N)$ if the proportion of time spent diving $(\omega_{d,t} )$ did not exceed 50% of the maximum possible diving time. Seals must surface to breathe; they spend a maximum of 88.8% of any 2 h time interval underwater (Russell et al. 2015; Carter et al. 2020). Thus,$Z_{t} = N$ where $\omega_{d,t} < 0.444$, corresponding to intervals where the majority of time is spent either at the haulout or at-sea at the surface. Next, any interval that was flagged as “unreliable” in data processing due to lack of location or tag summary data (grey seals: 10.9%; harbour seals: 9.3% of intervals) was assigned a known state of “unknown”$(Z_{t} = Unk)$. The remaining intervals (where $\omega_{d,t} \ge 0.444$ and thus $Z_{t} \ne \left\{ {N, Unk} \right\}$) were inferred as either ARS or transit($Z_{t} \in \left\{ {ARS, Tr} \right\}$) for the four-state model, or focussed ARS, broad ARS or transit($Z_{t} \in \left\{ {fARS, bARS, Tr} \right\}$) for the five-state model based on step lengths ($l_{t}$: the Euclidean distance travelled in a 2 h interval) and turn angles $(\varphi_{t} )$. In the four-state model, the ARS state was inferred from the distribution that captured lowest step lengths and lowest directional persistence (highest turn angles). In the five-state model, the focussed ARS state was inferred from the distribution that captured lowest step lengths and lowest directional persistence, and the transit state was inferred from the distribution that captured highest step lengths and highest directional persistence. The broad ARS state was inferred from the distribution that captured intermediate step lengths and directional persistence.

Following Carter et al. (2020), step length was assumed to be Gamma-distributed: $l_{t} |Z_{t} = z \sim Gamma \left( {\mu_{z} /\sigma_{z} ,\sigma_{z} } \right)$ with the state-dependent mean step parameter $\mu_{z} > 0$ and shape parameter $\sigma_{z} > 0$. A wrapped Cauchy distribution was assumed for turn angles:$\varphi_{z} |Z_{t} = z \sim wCauchy \left( {0,\gamma_{z} } \right)$ with mean 0 and state-dependent directional persistence parameter $0 < \gamma_{z} < 1$. The mean angle parameter was set to zero to maintain biological interpretability (Carter et al. 2020). Maximum likelihood (ML) estimation was performed using the forward algorithm and the most likely state sequence was decoded using the Viterbi algorithm (Zucchini et al. 2016). Starting values for state-dependent parameters must be specified, and inappropriate values may strongly influence the ability of the HMM to find the global optimum of the likelihood function, resulting in convergence issues or misleading outputs (Zucchini et al. 2016). Following guidelines from Michelot et al. (2016), 25 sets of random initial values were generated for state-dependent parameters between reasonable upper and lower bounds based on biological rationale, and HMMs were fitted per species with each set. The resulting 25 ML scores per species were inspected to ensure that the global optimum was achieved, and a model with optimal score was then retained. Model validation was performed by visual inspection of fitted distributions and pseudo-residuals.

### Foraging habitat association model

#### Data cleaning

State assignments from the best HMM were used as input data for foraging habitat association analysis (Objectives 2 and 3) following a number of further cleaning steps. Firstly, since seals may exhibit unrepresentative habitat associations for a short time as a response to being captured for tag application (McKnight 2012), any trip initiated within seven days of capture was excluded from the dataset. Seals may also undertake short trips (< 8 h) nearby the haulout, and although the associated movement patterns may resemble ARS, their behaviour, at least in part, is likely related to waiting for tidal haulouts to become available (Thompson 1989; Cordes et al. 2011). Therefore trips < 8 h in length were excluded from the dataset (3.9% and 6.1% of intervals for grey and harbour seals respectively). Next, following Carter et al. (2025), data were clipped to summer (May – September) for grey seals, and autumn–winter-spring (September – May) for harbour seals. This avoids the potential of including data related to breeding and moulting activity, which may confound inference of foraging behaviour. Data were then spatially clipped to the North Sea study region (ICES Area 4), excluding foraging trips outside of this area.

Trips were then assigned to one of six regions based on the location of the associated haulouts, as per Carter et al. (2025). Regions were designated on the basis of location and habitat composition, as well as species-specific similarities in movement patterns and diet (Carter et al. 2025). Trips that transitioned between regions (2.7% and 0.1% of trips for grey and harbour seals respectively) were divided at the midpoint, with the first half of the trip being assigned to the departure region, and the latter half assigned to the destination region. Next, locations with missing habitat covariate data (see below) were excluded (2.31% and 4.05% of grey and harbour seal locations respectively). Lastly, locations within in The Dollart (an embayment in the inner Ems estuary, Wadden Sea) were removed (0% of grey and 1.93% of harbour seal locations respectively). Harbour seals hauling-out in the Wadden Sea predominantly forage offshore, but 20% of the tagged individuals also spent large amounts of time at sea near haulouts within The Dollart. Unlike in other areas where some individuals may remain entirely within an embayment throughout the tracking period (e.g., The Wash, Southeast UK) and therefore must forage there, the behaviour of seals in The Dollart may be dominated by in-water resting (which may last for several days) between offshore foraging trips. Furthermore, the substrate in the Dollart is muddy, and thus distinct from foraging habitat encountered elsewhere. Including these data as putative ARS would therefore have a large, potentially spurious, impact on results. Analysis was repeated with these locations retained for comparison (Supplementary Information). The above cleaning and restrictions resulted in removal of a further 18 grey seals and one harbour seal from the dataset. The final datasets for habitat association models therefore comprised 3482 foraging trips from 116 grey seals, and 10,682 trips from 325 harbour seals (Table 1, Fig. 1).

**Table 1:** Final sample sizes of foraging trips and tracked seals by species and region. Since some seals made trips in multiple regions, the total number of unique trips and individuals is shown in parentheses

Region	Grey Seals		Harbour Seals	
	Trips	Ind	Trips	Ind
Orkney	655	15	411	17
Moray Firth	274	15	2284	61
East UK	483	25	286	9
Southeast UK	784	40	1978	53
Dutch Delta	594	25	1135	27
Wadden Sea	792	40	4600	168
Total	3582 (3482)	160 (116)	10,694 (10,682)	335 (325)

#### Statistical modelling

The HMM with two ARS states was preferred for both species (see Results), and the resulting state estimates were used as a binary response term in logistic regression models (e.g., Kane et al., (2020); Nykänen et al., (2025)). Specifically, a nested binomial approach was adopted to investigate: (i) the probability of ARS (either broad or focussed ARS: coded as 1) given encounter (transit coded as 0), and (ii) the probability of focussed ARS (coded as 1) given being in ARS (broad ARS coded as 0). Non-diving state intervals were excluded since at-sea surface activity is likely related to resting and digestion (Sparling et al. 2007), and not necessarily contingent upon a behaviour-specific habitat association. For each analysis, the binary response term was modelled per species as a function of covariates (see below) in a generalised additive mixed model (GAMM) using the “bam” function in the R package “mgcv” (Wood 2020), with a binomial error family and logit link function. An individual identifier for each seal was included as a random intercept term, ensuring that modelled relationships were not dominated by data-rich individuals (Gillies et al. 2006). GAMMs were used because of the ability to fit non-linear relationships to continuous covariates, and to leverage the low memory footprint of the “bam” function which is optimised for large datasets such as this (Wood 2017).

To address Objective 2 and determine regional differences in the prevalence of each scale of ARS, only region was considered as a covariate in the models described above. To address Objective 3 and determine foraging habitat associations, the response variable was modelled as a function of seabed geomorphology, substrate type, region, and, for grey seals, summer mean potential energy anomaly (PEA: a metric of stratification, representing the amount of energy required to result in complete mixing of the water column under typical conditions (Jones 2024)). PEA was only used in models for grey seals since waters are predominantly well mixed outside of summer in the months corresponding to harbour seal data. See below for more detail on covariate extraction. The full model featured a three-way interaction between categorical covariates (seabed geomorphology, substrate type and region). This allowed for the possibility that the importance of certain geomorphological features was mediated by their substrate type (i.e., sandy peaks represent different foraging habitat to rocky peaks), and that selection of such features may depend on regional differences in encounter rates and/or prey distribution. The full model for grey seals also featured summer mean PEA fitted in a cubic regression spline and “select” set to TRUE, such that, if uninformative, it could be penalised to zero (Wood 2017). This term was also fitted in an interaction with region, allowing a separate smoother for each region. The number of knots (*k*) was selected by trialling models with different values and selecting the one that minimised the AIC score while still returning a relationship that was biologically interpretable. For comparability with harbour seals, models without PEA were also fitted for grey seals (see Supplementary Information).

Models were simplified using backwards model selection by AIC score (threshold for covariate removal: ∆AIC ≤ 2 (Burnham and Anderson 2002)). Model residuals were examined for evidence of serial autocorrelation and no issues were detected. Model predictions (mean and associated upper and lower 95% confidence intervals) were generated using a posterior sampling approach with 1000 random draws (Wood 2017). The output from this posterior sampling process for analysis (i) was multiplied by the output for analysis (ii) to get the overall probability of focussed or broad ARS, propagating the probability of ARS given encounter and associated uncertainty. To investigate any possible influence of uncertainty in state assignments on the results, estimated state probabilities obtained from the HMM were used in a multinomial draw, returning a new possible state sequence which was then used to code the response term and refit the GAMM following previous studies (Kane et al. 2020; Nykänen et al. 2025). This process was repeated 100 times and model predictions for each iteration were compared to each other. No deviation from the original results was evident.

#### Habitat covariates

Habitat covariates were chosen on the basis of biological relevance to seals and/or their prey, and their availability for the study area as a whole. Static habitat covariates (including static representations of dynamic processes) were used in favour of dynamic covariates since data were pooled across years. For rationale relating to covariates, see Supplementary Information. Although previous research has shown distance to haulout to be an important factor in habitat selection in use-availability habitat models for grey and harbour seals (Aarts et al. 2008; Carter et al. 2022), it was not considered here as it is more related to habitat accessibility than foraging habitat selection. Water column depth was also excluded as both species are capable of diving to the seabed throughout the study area, and it is therefore unlikely to be a key determinant of ability to forage. Moreover, distance to haulout and depth were excluded since they may be correlated with other more ecologically relevant covariates, masking their effect. A main objective of this study was to investigate the characteristics of seal foraging habitat rather than create an optimal model for prediction of seal distribution, as in Carter et al. (2025).

Since each ARS or transit location was assigned to the midpoint of a 2 h time interval (see “Telemetry Data Processing” above), it was desirable to capture the dominant habitat type experienced during each time interval, rather than simply extracting covariate values underlying the midpoint. A series of interpolated points were therefore generated at 1 min intervals along the original seal track. Values were then extracted for each covariate at each of these 1 min intervals. All interpolated points that fell within a 2 h interval corresponding to the ARS or transit location were then summarised to the median for PEA, and mode for seabed geomorphology and substrate. All processing and extraction of environmental covariates was done using the “terra” (Hijmans 2023) and “sf” (Pebesma and Bivand 2023) R packages.

## Results

### Objective 1: Presence of multi-scale ARS

**Fig. 2:**
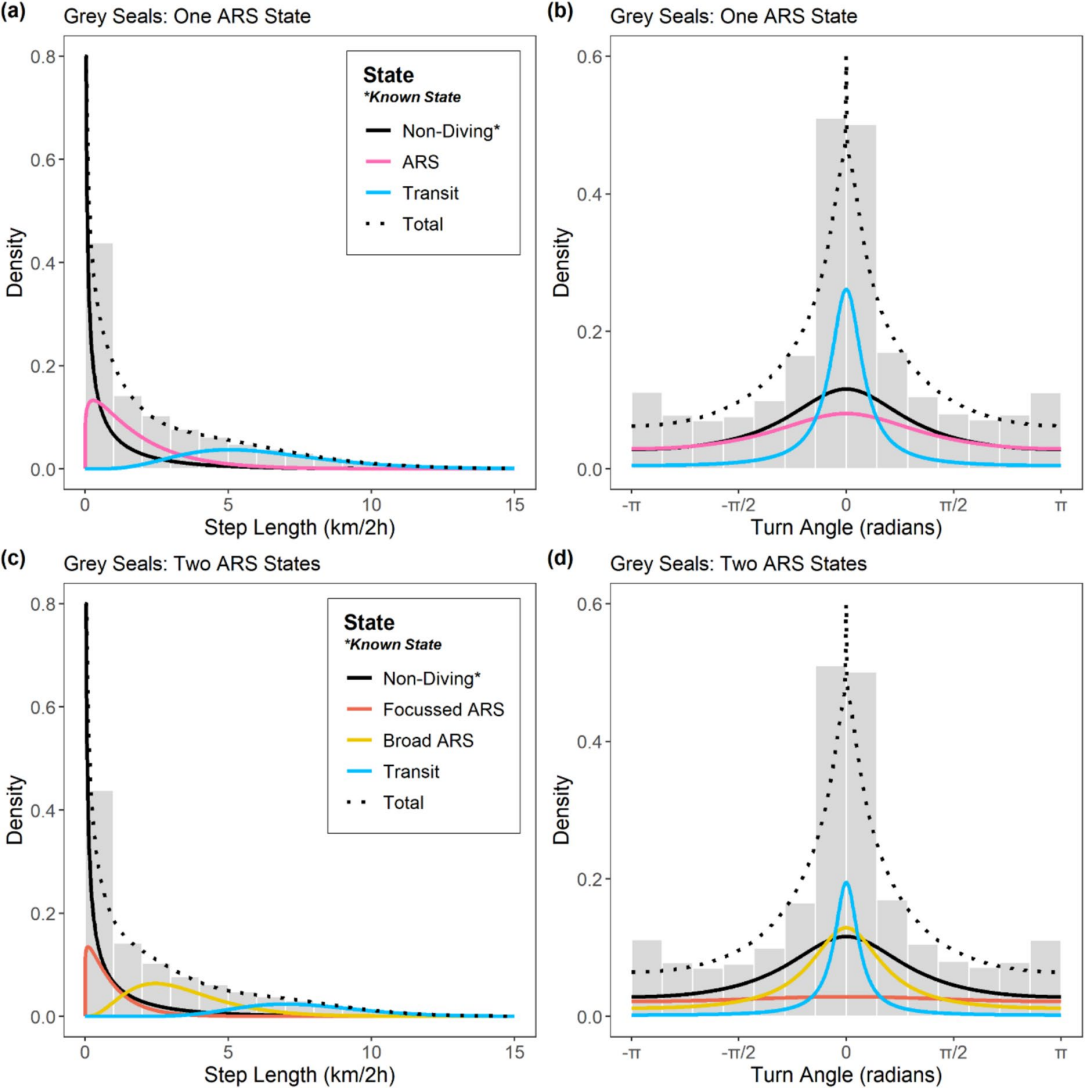
State-dependent distributions for step length and turn angle for HMMs with (**a**–**b**) one ARS state, and (**c**–**d**) two ARS states for grey seals. Dashed line shows the marginal distribution. To aid interpretation, distributions for “unknown” state are omitted since this state has no biological relevance

**Fig. 3:**
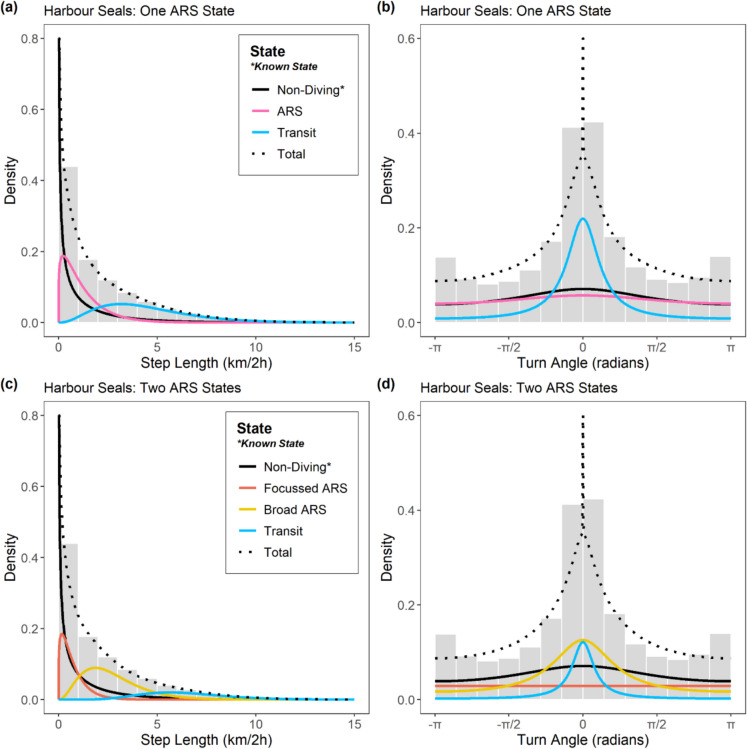
State-dependent distributions for step length and turn angle for HMMs with (**a**–**b**) one ARS state, and (**c**–**d**) two ARS states for harbour seals. Dashed line shows the marginal distribution. To aid interpretation, distributions for “unknown” state are omitted since this state has no biological relevance

**Fig. 4:**
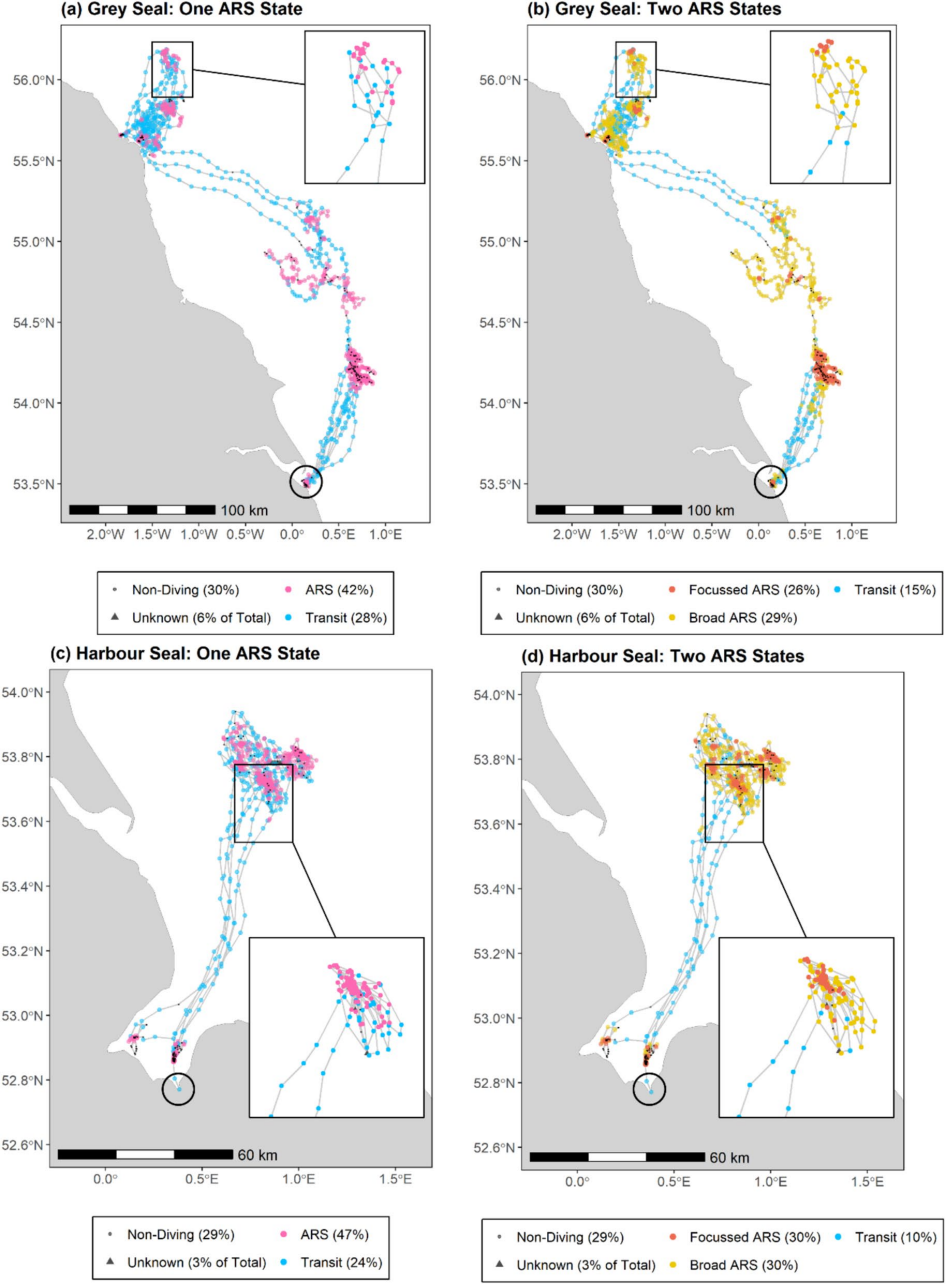
Example tracks for (**a**–**b**) a grey seal and (**c**–**d**) a harbour seal tagged in Southeast UK, with state assignments from HMMs with (**a**, **c**) one ARS state, and (**b**, **d**) two ARS states. Large open circles denote tagging locations. Percentages show the activity budgets (excluding “unknown” state intervals; unknown state is expressed as a percentage of all intervals). Inset magnified plots show the track with state assignments for a portion of one trip

For both species, there was strong support for the five-state HMM (two ARS states) over the four-state HMM (one ARS state). The five-state model was vastly superior according to BIC (ΔBIC: -26,421 & -24,616 for grey and harbour seals respectively). Inspection of fitted distributions (Figs. 2–3) and mapped state assignments (Fig. 4) revealed that the five-state model provided more biologically plausible outputs. The four-state model potentially underestimated ARS and over-estimated transit, since the transit distribution captured some slow tortuous movements. In particular, there was strong overlap in state-dependent distributions for step lengths between the ARS and transit states in the 4-state model, particularly for harbour seals (Fig. 3a). In contrast, the five-state model clearly distinguished focussed and broad ARS for both species, with the transit distribution being more restricted to faster, directed movements.

Across the entire dataset, the five-state model estimated 10.02% and 17.13% more ARS intervals than the four-state model for grey and harbour seals respectively. At the individual level, for grey seals the five-state model estimated on average 9.91% more ARS than the four-state model (median among individuals, IQR: 7.13–13.77%), up to a maximum of 25.2%. For harbour seals, the five-state model estimated on average 16.30% more ARS (IQR: 11.23–22.66%), up to a maximum of 46.38%. All seals exhibited both ARS states, although for six grey and nine harbour seals, focussed ARS comprised < 10% of putative foraging activity. Broad ARS comprised at least 18% of putative foraging for grey seals, while two harbour seals spent < 10% of putative forging in broad ARS.

There were subtle differences between the species in the characteristics of each state. The state-dependent mean step length parameters for fitted distributions were greater for grey than harbour seals in each state, indicating faster movements (focussed ARS: 0.09 (0.09 SD) vs 0.07 (0.06 SD); broad ARS: 3.38 (1.76 SD) vs 2.70 (1.50 SD); transit: 7.63 (2.18 SD) vs 6.41 (2.19 SD)). Similarly, the turn angle concentration parameter was greater for grey than harbour seals in each state, indicating greater directional persistence (focussed ARS: 0.07 vs < 0.01; broad ARS: 0.53 vs 0.46; transit: 0.81 vs 0.76).

**Fig. 5:**
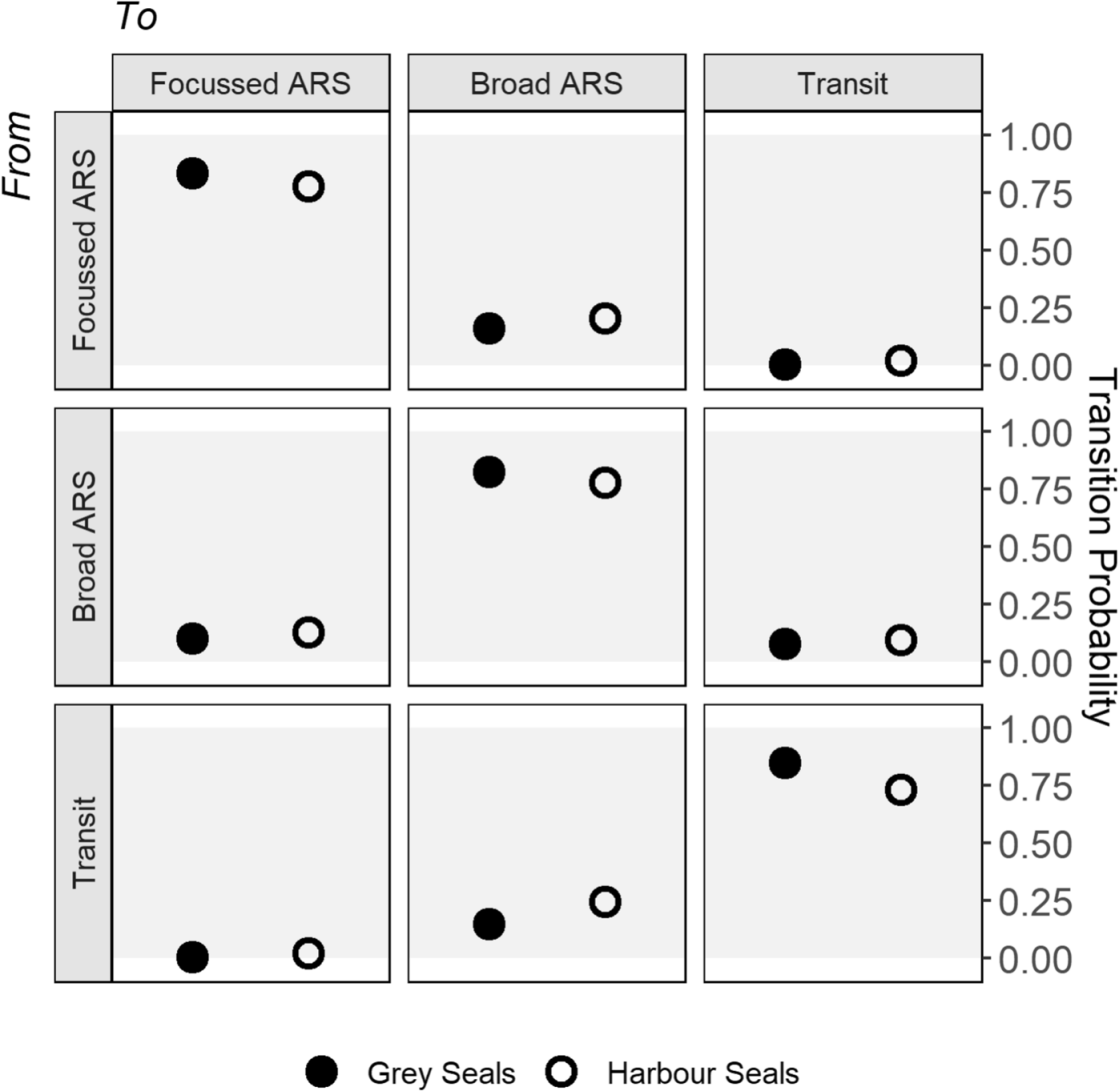
State transition probability matrix for transit and ARS states from five-state HMMs fitted to grey (closed symbols) and harbour seals (open symbols)

According to state transition probabilities, the probability of remaining in each state was high for both species (grey seals: ≥ 0.82, harbour seals: ≥ 0.74) (Fig. 5, Supplementary Information Table S1). The probability of switching from transit into broad ARS was greater than into focussed ARS for both species (grey seals: 0.14 vs 0.004, harbour seals: 0.25 vs 0.03). Likewise, the probability of switching into transit was greatest from broad ARS for both species (grey seals: 0.08 vs 0.005, harbour seals: 0.1 vs 0.02). The probability of switching between ARS states was greatest for focussed to broad ARS (grey seals: 0.16 vs 0.10, harbour seals: 0.21 vs 0.13).

### Objective 2: Regional differences in multi-scale ARS

**Fig. 6:**
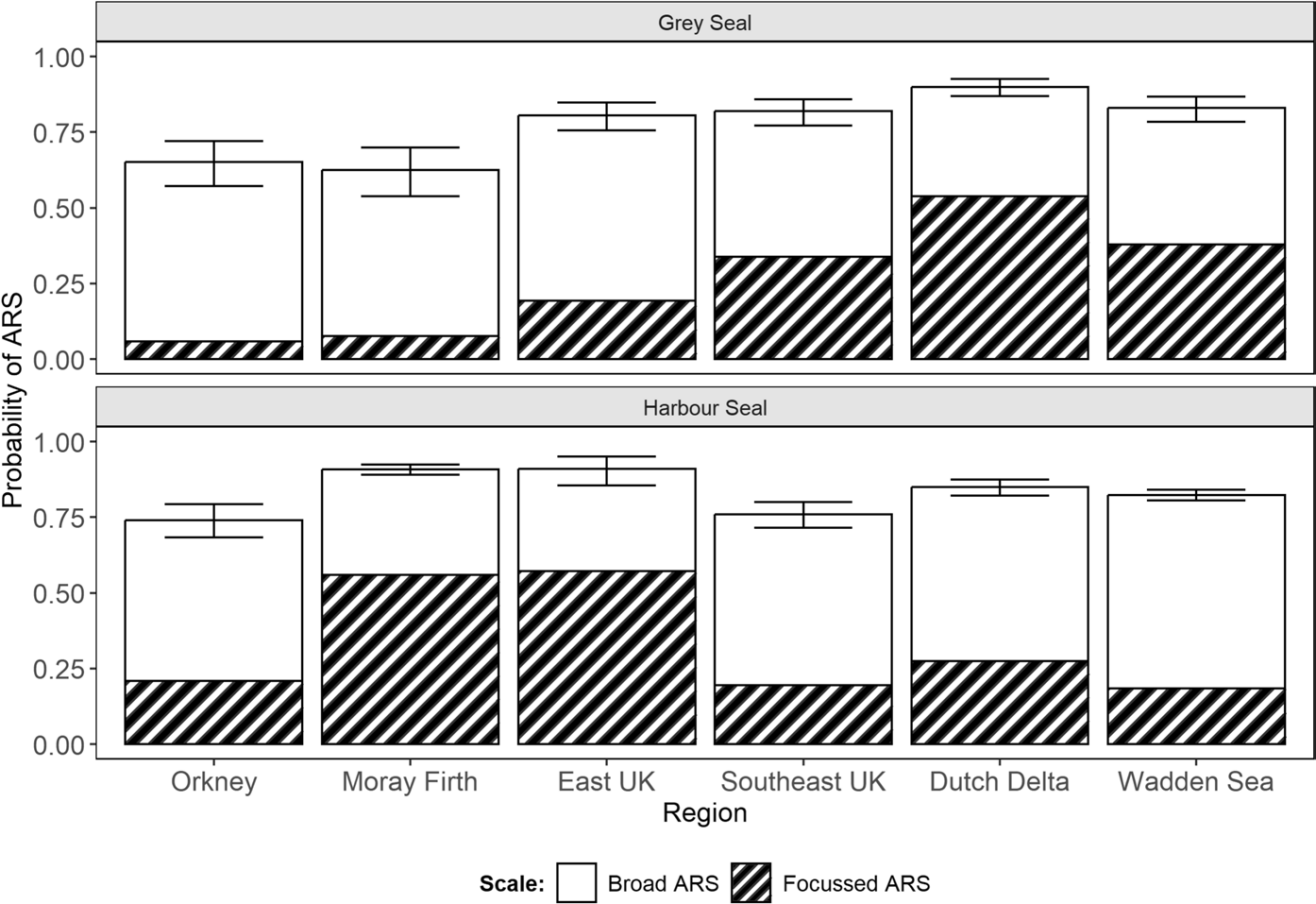
Regional differences in the overall probability of broad (open bars) and focussed ARS (hatched bars) for grey and harbour seals. Error bars show 95% confidence intervals around the mean probability of any ARS (broad or focussed) given encounter. The remaining probability space (up to 1) reflects the probability of being in transit state

Foraging habitat association models fitted with just region as an explanatory term revealed species-specific regional differences in the probability of ARS at both broad and focussed scales (Fig. 6). For grey seals hauling-out in all regions except for the Dutch Delta, the probability of broad ARS (range: 0.44–0.61) exceeded that of focussed ARS (range: 0.06–0.38) (Fig. 6). Grey seals making foraging trips from haulouts in the Dutch Delta recorded a particularly high probability of focussed ARS (0.54) compared to other regions, while those in Orkney, the Moray Firth, and East UK recorded a particularly low probability of focussed ARS (≤ 0.19). For harbour seals, broad ARS was again the dominant foraging state (range: 0.53–0.64), except for among seals hauling-out in the Moray Firth and East UK (range: 0.34–.35), where the overall probability of focussed ARS was higher (range: 0.56–0.57) than elsewhere (range: 0.18–0.27) (Fig. 6).

### Objective 3: Habitat associations of multi-scale ARS

For both grey and harbour seals, the probability of being in an ARS state (broad or focussed) given encounter, and the probability of focussed ARS given being in ARS, was best explained by the full model (Supplementary Information Table S2). Thus, both species demonstrated region-specific selection of different seabed habitats (defined by an interaction between region, geomorphology and substrate type), and for grey seals, the best model also included a region-specific relationship with water column stratification (PEA).

**Fig. 7:**
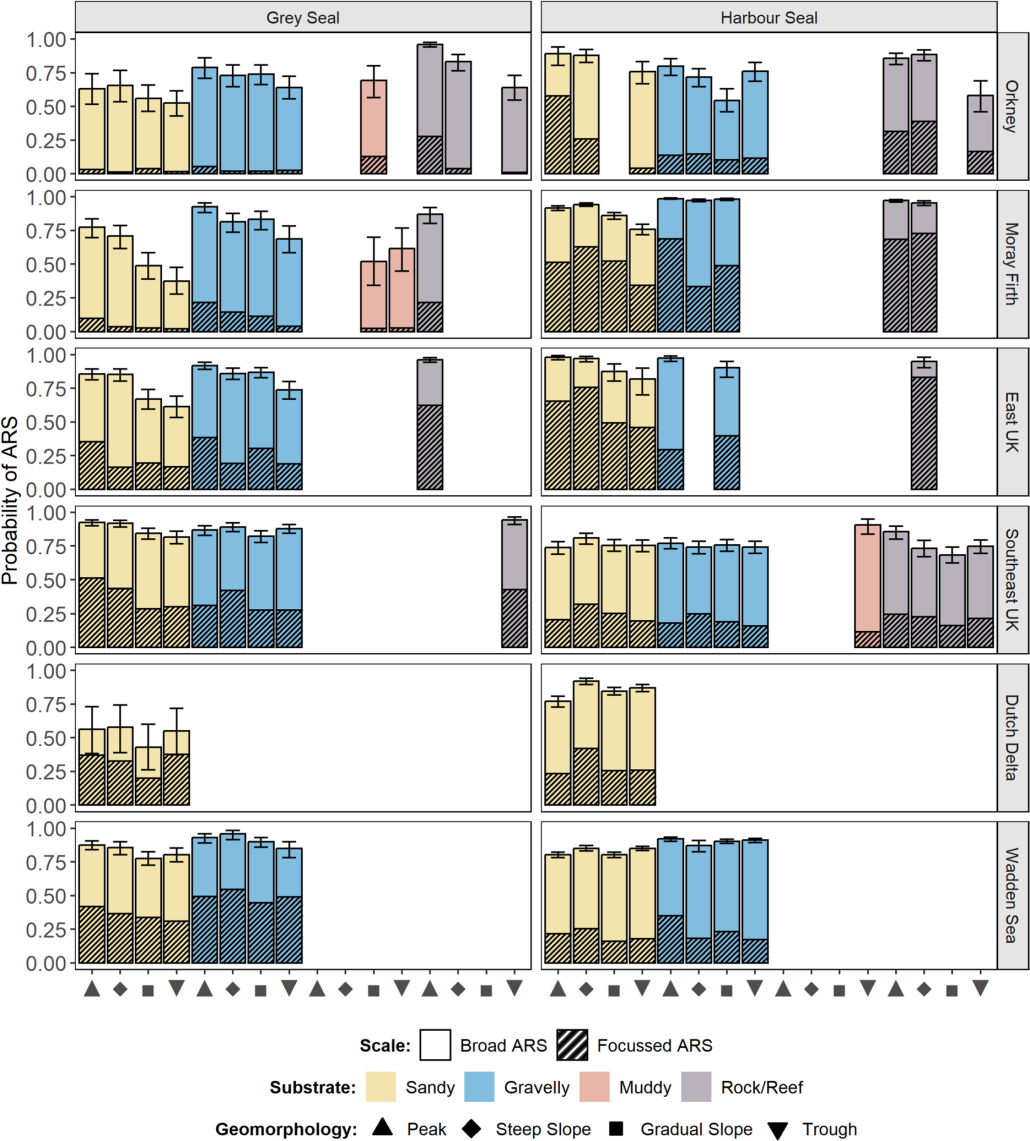
Regional differences in seabed foraging habitat associations for grey (left) and harbour seals (right). Stacked bars show the mean probability of focussed ARS (hatched) and broad ARS (open) given encounter per habitat type. The sum of the two scales gives the overall probability of any ARS. The remaining probability space (up to 1) reflects the probability of being in transit state. Error bars show 95% confidence intervals around the mean probability of any ARS. Colours denote different substrate types, shapes denote different geomorphologies. Habitat types that were rarely encountered (< 100 observations) are not shown

Both grey and harbour seals exhibited regional differences in which seabed habitat types were associated with the highest probability of ARS at either focussed or broad scales (Fig. 7). In general, peaks and steep slopes were the geomorphologies most likely to be associated with ARS, followed by gradual slopes then troughs, although this trend was clearer for grey than harbour seals. The relative importance of different features was mediated by substrate type. For grey seals, gravelly features were generally associated with a higher probability of ARS than the same geomorphologies in sandy substrates. For grey seals hauling-out in Orkney and East UK, rocky/reef peaks were associated with a particularly high probability of focussed ARS compared to other features (Fig. 7). For harbour seals, although the overall probability of ARS was comparable between substrate types in most regions, for seals hauling-out in Orkney and East UK, the probability of focussed ARS was far greater in association with sandy and rocky/reef habitats than gravelly habitats. Muddy habitats were not frequently encountered by either species (although see Supplementary Information Fig. S5 for results relating to harbour seals in The Dollart, Wadden Sea). Where they were encountered, grey seals showed a relatively low probability of ARS (Fig. 7). Muddy troughs were associated with a particularly high probability of broad ARS for harbour seals hauling-out in Southeast UK, although this result was associated with a relatively small (< 200) number of observations.

**Fig. 8:**
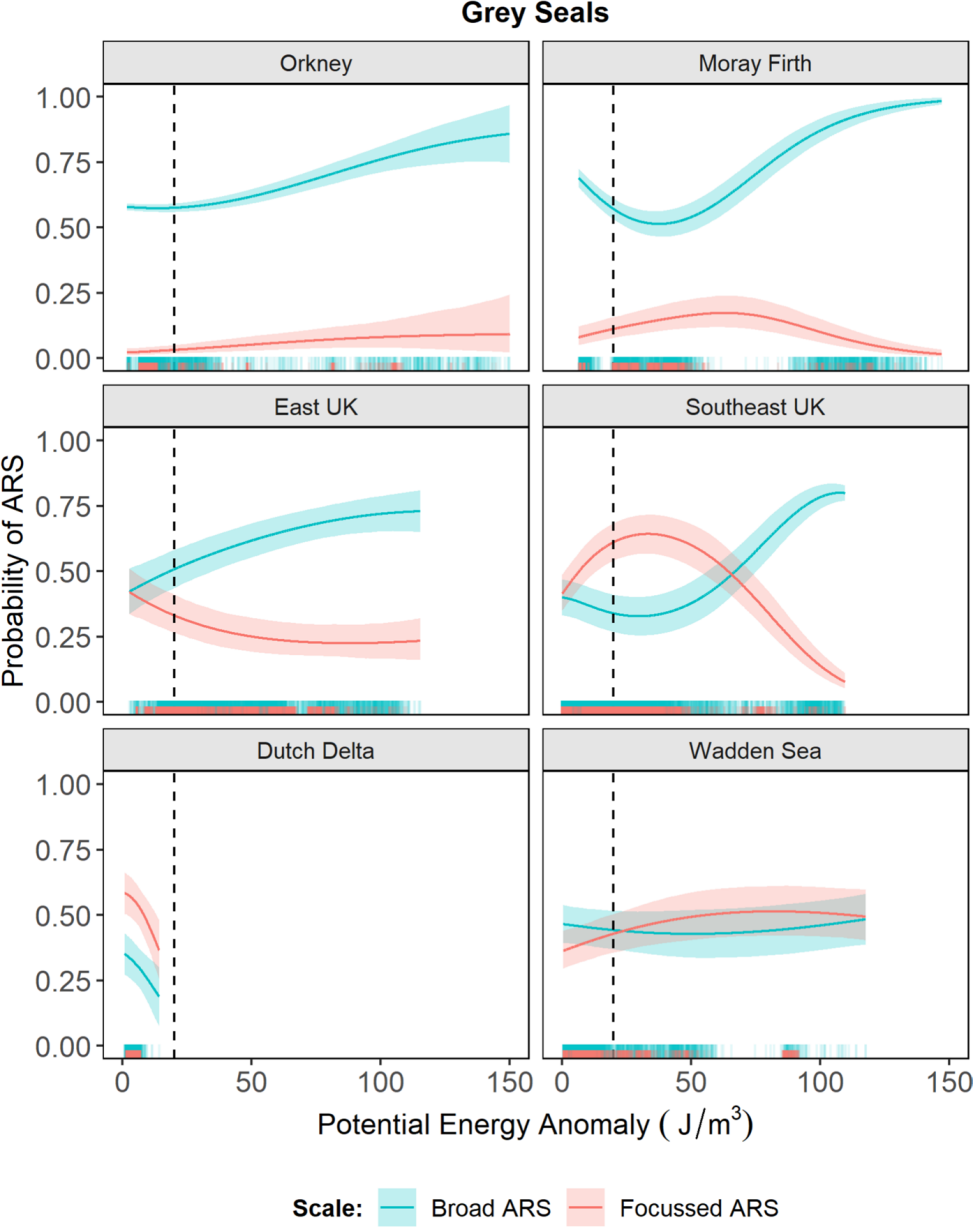
Regional differences in water column foraging habitat associations for grey seals. Lines show the mean predicted probability of broad ARS (blue) and focussed ARS (red) under increasing water column stratification. Shaded areas show 95% confidence intervals around the mean. The sum of the two scales gives the overall probability of any ARS. Rugs show PEA values where ARS occurred, coloured by scale. Dashed line shows the approximate boundary between mixed and stratified waters (i.e., frontal areas)

In addition to seabed habitat features, the probability of ARS was also influenced by region-specific responses to water column stratification for grey seals (Fig. 8). The probability of broad ARS generally increased with increasing stratification, except for in the Dutch Delta where a negative relationship was returned (although the range of PEA values encountered was limited to 0–20 J/m^3^). In Southeast UK, the probability of focussed ARS showed a strong peak for values between 20 and 40 J/m^3^ PEA, followed by a sharp decline, suggesting that this behaviour was associated with frontal areas and adjacent stratified water. In East UK, the probability of focussed ARS declined with increasing PEA, suggesting that this behaviour was more likely in mixed than stratified waters. Models fitted without PEA for grey seals returned comparable results for seabed habitat types to those presented here, except for in the Dutch Delta where the probability of ARS was higher for each habitat type at both scales (Supplementary Information Fig. S6). This suggests that the negative trend with increasing PEA in the Dutch Delta had a negative effect on the baseline probability of ARS reflected in Fig. 7.

## Discussion

To our knowledge, this study is the first to use HMMs to investigate ARS at multiple spatial scales from animal tracking data in any species. The modelling approach can be easily applied to other taxa to investigate scale-dependent foraging behaviour; a phenomenon that has been overlooked for many species. Our analysis provides evidence for scale-dependent foraging in two sympatric marine top predators; grey and harbour seals. ARS behaviour can be distinguished for both species at two spatial scales: broad extensive and more spatially focussed ARS, and the characteristics of these behaviours differ between the two species (Objective 1). Furthermore, the dominance of one scale over another varies among individuals and across regions in a species-specific manner (Objective 2). Finally, the habitat associations of ARS vary regionally across the seascape, and the influence of different habitat features on foraging decision making can be scale-dependent (Objective 3).

While hierarchically structured HMMs have been applied to animal-borne biologging data to investigate behaviours occurring at different temporal scales (Leos-Barajas et al. 2017), until now the potential for HMMs to quantify ARS at multiple spatial scales from such data has been overlooked. Indeed, previous HMM studies on grey and harbour seals have either used complete pooling to maximise statistical power but assumed one scale of ARS (e.g., McClintock et al. (2013)), or acknowledged that the characteristics of ARS may vary among individuals by fitting individual models with one ARS state, but lacked the ability to draw population-level comparisons of state characteristics (e.g., Russell et al. (2015)). By using a complete pooling approach but explicitly accounting for scale, this study allows for the possibility of variation among individuals in both the existence and prevalence of different ARS scales. Comparison with more conventional HMMs allowing one ARS state revealed that such models are likely overly simplistic; the transit state frequently captures slow tortuous movements that resemble ARS (Figs. 2–4). Among individuals, the conventional model estimated on average 9.91% (range: 0.62–25.16%) and 16.30% (range: 2.73–46.38%) fewer foraging intervals for grey and harbour seals, respectively. The implication of not accounting for this plasticity in scale, particularly when models are fitted at the population-, rather than individual level, could therefore be underestimation of foraging activity, leading to inaccurate activity budgets and misrepresentation of important foraging areas. This could result in suboptimal outcomes for spatial conservation management where inference from HMMs is used to designate protected areas or inform marine spatial planning.

Results show that all tracked seals of both species exhibited both broad and focussed ARS. This could be consistent with seals using a continuum of ARS scales, or multiple ARS states which they transition between while foraging. The fact that bouts of focussed ARS are most commonly preceded and followed by bouts of broad ARS, evidenced by the very low probability (close to 0) of any switching between transit and focussed ARS states (Fig. 5), suggests that seals employ a nested-scale foraging strategy. Seals may therefore initiate ARS at a broad spatial scale upon encountering prey spillover or environmental cues relating to profitable foraging areas, before switching to a more focussed search upon encountering denser prey patches. Seals might then switch back to broad ARS when prey patches become depleted, or competition is high, and individuals are compelled to leave a patch but have not reached satiation. Such nested-scale ARS strategies have been well documented in flying seabirds and appear to be associated with higher prey encounter rates than single-scale strategies (Pinaud and Weimerskirch 2005, 2007; Pinaud 2008; Hamer et al. 2009). We hypothesise that nested-scale foraging strategies may be particularly prevalent in species that feed on cryptic or dispersed prey, relying on environmental cues rather than visual detection (which may elicit a more abrupt shift in movement scale). We recommend further research to test this hypothesis among different feeding guilds. Other state-space approaches such as continuous state models (Jonsen et al. 2019) and HMMs with covariates influencing state-dependent parameters (Carter et al. 2020) may be useful to further examine the continuum of ARS scales. We elected to use a more conventional HMM framework here to allow the estimation of tangible states (and incorporation of known states), facilitating comparison to previous studies featuring one scale of ARS, and to highlight the broad accessibility and applicability of this approach for other taxa.

Although both ARS scales were exhibited by all individuals, there were individual and regional differences in their prevalence. Such variation may indicate differences in target prey species, which could be driven by prey abundance and/or interference competition. Optimal foraging theory dictates that tortuosity should decrease, and step length increase when prey becomes more dispersed (Ritchie 1998; Bartoń and Hovestadt 2013). Thus, broad ARS may be related to targeting prey species that are dispersed in the environment (e.g., gadids), compared to denser more clumped distribution of prey species such as sandeels (*Ammodytes spp.*) and some flatfish species which likely aggregate in more discrete habitats (Holland et al. 2005; Hinz et al. 2006). Indeed, sandeels and flatfish are particularly prevalent in the diet of harbour seals hauling-out in the Moray Firth and East UK regions (Wilson and Hammond 2019) where focussed ARS is more dominant than in other regions (Fig. 6). In general, broad ARS was the dominant search scale for both species, with the exception of the Dutch Delta region for grey seals, and East UK and Moray Firth regions for harbour seals (Fig. 6). Indeed, for six grey and nine harbour seals, < 10% of putative foraging activity was assigned to the focussed state. This suggests that although seals likely do focus search activity at discrete known foraging grounds, a large part of their activity is dedicated to searching for prey over wider spatial areas or perhaps foraging opportunistically while moving between foraging grounds. To further understand the relative costs and benefits of scale-dependent search behaviour and its biological significance, future studies should aim to combine movement metrics with measures of foraging success from ancillary data, such as physiological indicators of feeding (Carter et al. 2016).

Beyond scale-dependent foraging strategies, sympatric species with different body sizes often exhibit interspecific differences in search scale as a mechanism to avoid competition while foraging (Jarman and Sinclair 1979; Dorfman et al. 2022). Allometric scaling of foraging behaviour has been posited as a niche dimension over which terrestrial herbivores with overlapping dietary niches can partition resources, with larger species searching over greater spatial scales (Laca et al. 2010). Although this has not been tested experimentally for sympatric marine predators to our knowledge, our results suggest some support for this hypothesis in seals. Grey seals are larger in size than harbour seals; the nose-tail length of individuals included in this study was significantly greater for grey seals (median:166 cm; IQR: 149–177) compared to harbour seals (median: 140 cm; IQR: 131–145) (Wilcoxon Rank-Sum Test: *W* = 28,670, *p* < 0.001). There were subtle differences in the characteristics of putative ARS behaviours between the species, with the HMM state-dependent mean step lengths being marginally greater for grey then harbour seals for both broad and focussed ARS (Figs. 2–3). The turn angle concentration parameter was also lower for harbour than grey seals for both ARS states, suggesting more spatially restricted behaviour. Whether or not these differences hold tangible biological relevance requires further investigation. Besides interspecific differences in body size, size varies with age, and grey seals exhibit sexual size dimorphism. Investigation of age and sex effects was beyond the scope of this study but should be a future priority, particularly as young grey seals overlap in size (and perhaps niche) with adult harbour seals. Examination of search scale through time for both species in areas where harbour seal numbers are depleted or declining may offer valuable insights into potential at-sea drivers of population trajectories.

By combining outputs from HMMs with habitat association models in a use-encounter framework, this study was able to quantify the relative probability of foraging at different habitat features across spatial scales. The results demonstrate marked regional differences in the foraging habitat selection of both grey and harbour seals across the North Sea seascape (Figs. 7–8). Previous research has demonstrated regional differences in overall habitat selection for both species in the UK and Ireland (Carter et al. 2022), but this has not yet been examined in the context of putative foraging behaviour. Our results further demonstrate the utility of geomorphological seabed classification for characterising important seal habitat, supporting the findings of Wyles et al. (2022). For example, we find that peaks and steep slopes are associated with relatively high probability of ARS compared to gradual slopes and troughs in most regions (Fig. 7).

Overall, within a region, the relative importance of different seabed habitat features for focussed ARS reflected the relationships for ARS in general (Fig. 7), suggesting that the two scales may not always represent targeting of different prey species. However, there were region- and species-specific instances of scale-dependent habitat selection. For example, for harbour seals hauling-out in Orkney, the probability of ARS (broad or focussed) at peaks and steep slopes was comparable across sandy, gravelly and rocky/reef substrates. Yet sandy peaks were associated with a far greater probability of focussed ARS than other features. This may be related to targeting tightly aggregated prey species such as sandeels, which are prevalent in the diet of harbour seals in Orkney (Wilson and Hammond 2019). Similarly for grey seals in Southeast UK, the probability of ARS in general was comparable for peaks and steep slopes in both sandy and gravelly substrates. However, the probability of focussed ARS was greater for steep slopes than peaks in gravelly areas, and for peaks than steep slopes in sandy areas (Fig. 7). This suggests that seals can employ different search strategies in different habitat types, possibly related to habitat-specific differences in prey types or prey densities.

In addition to seabed features, there was also a scale-dependent relationship with water column stratification for grey seals in some regions. With the exception of the Dutch Delta and Wadden Sea where waters are predominantly well mixed, grey seals hauling-out in all other regions showed differential relationships with stratification for broad compared to focussed ARS (Fig. 8). Broad ARS was most associated with high PEA values, representing stratified waters. The probability of focussed ARS generally declined with strongly stratified conditions but was particularly high in the Southeast UK region in association with PEA values of 20–40 J/m^3^ (Fig. 8). These values likely correspond to oceanographic fronts where mixed and stratified waters meet, and stratified waters adjacent to the fronts (Supplementary Information Fig. S3). This suggests a more focussed search strategy being employed in areas associated with the Flamborough tidal mixing front; a seasonally persistent feature that extends from the east coast of England eastwards to the fringes of the Dogger Bank (Hill et al. 1993). Such areas serve to aggregate prey, providing predictable foraging opportunities for predators (Cox et al. 2018). This study used static seabed features and static representations of dynamic processes as covariates to allow population-level comparison of habitat selection at the seascape scale. However, for future studies of individuals at finer spatial scales, in-situ measurements of environmental conditions from animal-borne tag sensors may provide further insight into the mechanistic relationships underpinning foraging habitat selection (Nowak et al. 2020).

This study fills an important knowledge gap in quantifying the influence of environmental features on grey and harbour seal foraging decisions at multiple spatial scales. It benefits from a large sample size, covering key centres of abundance for both species, allowing seascape-scale analysis of behaviour. Moreover, the results further demonstrate the importance of considering regional variation in habitat associations, as outlined in Carter et al. (2022), to fully understand environmental drivers of behaviour and distribution. However, the imbalance in sample size among the regions should be held in mind when interpreting results, particularly for harbour seals in the East UK region where the number of tracked individuals was far below that of other regions (Table 1). Ideally, future studies should aim to track more seals in this region, however the catastrophic population decline recorded here in recent years (Thompson et al. 2019) may be prohibitive. Seasonality should also be considered when comparing results between the species. Differences in behaviour could be partially explained by the fact that grey seal data relate to summer, and harbour seals to autumn–winter-spring, and thus the two species may encounter different prey landscapes.

While the aim of this study was to generate population-level inference, it should be acknowledged that certain behaviours may not be well represented in the results. For example, due to lack of covariate data in coastal waters, putative foraging close to the coast is under-represented in the habitat association analysis for some regions. This is particularly the case for harbour seals in the Wadden Sea where environmental data were lacking inside tidal estuaries. Another consideration is that trips < 8 h duration were excluded due to the possibility that movement patterns resembling ARS are likely to be caused by seals waiting in the water for tidal haulouts to become available rather than by foraging. Seals may also wait in the water for the same reason at the end of a longer foraging trip if they arrive at the haulout before it is exposed, and this activity was retained in the study. We also excluded data in The Dollart where many harbour seals exhibit ARS-like behaviour between offshore foraging trips (Supplementary Information Fig. S4). While this behaviour may represent foraging, it may alternatively be related to in-water resting before returning to the wider North Sea to forage. Including such data can have a large impact on results (Fig. S5), thus future studies should aim to integrate direct metrics of foraging activity such as animal-borne video or prey catch attempts from accelerometers (e.g., Vance et al. (2021); Iorio-Merlo et al. (2022); Wynn-Simmonds et al. (2024)) to elucidate the nature of at-sea behaviour near the haulout and ground-truth inference from HMMs. Indeed, such data could also help to understand the biological significance of both scales of ARS.

In addition to behaviours that remain poorly understood, individual seals may exhibit particular foraging specialisations that were not accounted for in this study. For example, some individual grey and harbour seals have been shown to concentrate foraging activity at offshore energy structures, such as oil and gas platforms, pipelines and wind turbines (Russell et al. 2014). While the HMM formulation used here allows such individualistic behaviour to be captured, analysis of the influence of anthropogenic habitat features on behaviour was beyond the scope of this study. However, the results presented here provide a baseline understanding of seal associations with natural habitat features, upon which further analyses can add complexity. Future work should therefore aim to build on these outputs to capture the relative importance of novel anthropogenic foraging habitats to individuals and quantify the influence of specific structures on seal behaviour, given the underlying influence of natural environmental features.

## Conclusions

This study provides critical new information about the nature and habitat associations of foraging behaviour in two otherwise well-studied sympatric marine predator species. Our results provide evidence that both grey and harbour seals employ ARS at nested scales, a phenomenon that is largely understudied in marine predators other than seabirds. Moreover, we reveal individual and regional plasticity in the prevalence of both scales, and highlight that conventional analyses with one scale of ARS may underestimate foraging by up to 25% and 46% for grey and harbour seals respectively. We highlight that overlooking multi-scale ARS could therefore lead to inaccurate activity budgets and suboptimal outcomes where inference of important foraging areas is used to inform marine spatial planning and conservation management. Our study also reveals that grey and harbour seals exhibit regional differences in scale-dependent foraging habitat associations across the North Sea, suggesting that they employ different strategies for different target prey species or in response to different prey densities. Future studies should build on these findings, investigating the presence of multi-scale foraging in other aquatic predators and integrating proxies of foraging success to elucidate the relative costs and benefits of different search strategies.

## Supplementary Information

Below is the link to the electronic supplementary material.

Supplementary file1 (DOCX 2325 KB)

## Data Availability

The datasets analysed during the current study can be requested from the corresponding authors.
