# Supplementary material for: Scale-dependent foraging behaviour and habitat associations of two sympatric marine top predators
Source: Landsc Ecol. 2025 Dec 27;41(2):21. doi: 10.1007/s10980-025-02281-z (PMC12811352; doi:10.1007/s10980-025-02281-z)
Supplement: Supplementary file 1 — Supplementary file1 (DOCX 2325 KB) [file 10980_2025_2281_MOESM1_ESM.docx]

**Supplementary Information for Carter et al.:**

**Scale-dependent foraging behaviour and habitat associations of two sympatric marine top predators**

1. **Environmental Covariates**

*Seabed Substrate Type*

Seabed substrate type has been shown to influence the habitat selection of grey and harbour seals in the North Sea (Aarts et al., 2008; Carter et al., 2022), thus substrate type was extracted from the EMODnet Broad-Scale Habitat Map for Europe (EMODnet Seabed Habitats, 2021). Values were grouped into the following categories based on the dominant substrate type: gravelly, sandy, muddy, and rock/reef (including biogenic reef habitats such as mussel beds) (Fig. A1.6).

*
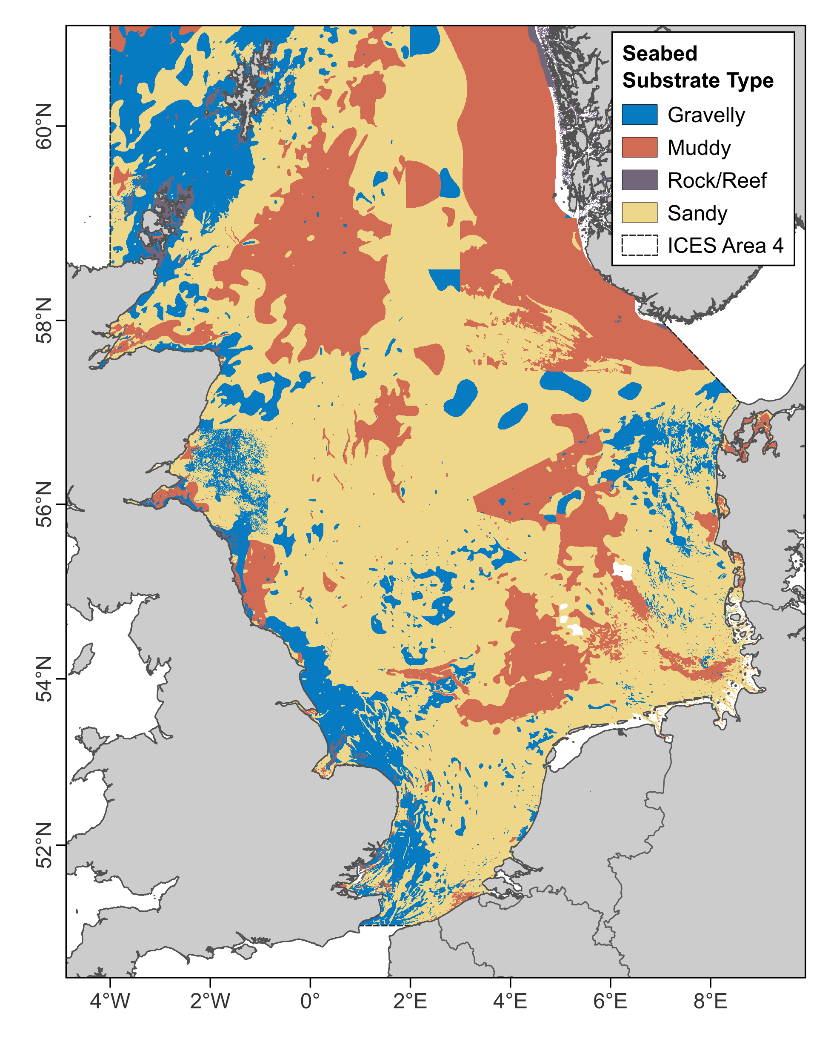
*

**Fig. A1**: Seabed substrate type for the North Sea study area (ICES Area 4). Data taken from the EMODnet Broad-scale Seabed Habitat Map for Europe (EMODnet Seabed Habitats, 2021).

*Seabed Geomorphology*

Seabed geomorphology is known to influence the behaviour of seals in the North Sea (Wyles et al., 2022). A raster of geomorphological features for the North Sea was generated from the EMODnet digital terrain model (DTM) for European sea regions (EMODnet Bathymetry, 2022). The bathymetric data were processed using the “r.geomorphon” extension for the Geographic Resources Analysis Support System (GRASS GIS) (Neteler & Mitasova, 2007) developed by Jasiewicz and Stepinski (2013), as described in Wyles et al. (2022). The raster was generated at a scale of 1/16 arc minutes (~115 m) corresponding to the resolution of bathymetric data (EMODnet Bathymetry, 2022), with a search radius of 450 cells (skipping the first 16) and a flatness threshold of 0.03 degrees (for detailed explanation of these parameters, see Jasiewicz and Stepinski (2013) and Wyles et al. (2022)). The parameterisation allowed for identification of both fine-scale (~115 m) as well as broad-scale (~100 km) features (e.g., both narrow and broad valleys). Feature types were initially grouped into three categories: peaks (comprising summits, ridges and shoulders), slopes (comprising slopes, spurs, hollows and relatively flat areas) and troughs (comprising footslopes, valleys and depressions). Since the slope category does not include information on steepness, this category was split into “steep” and “gradual” slopes post-hoc by applying a rugosity function to the bathymetric data and classifying any slope feature with a rugosity value above or below 1.5° as “steep” or “gradual” respectively. The threshold was determined using a mixture distribution model approach with the threshold value representing the upper 95^th^ percentile of the lower (gradual slope) distribution. Steep slopes therefore relate to areas with rapid change in seabed depth such as the fringes of peaks and troughs, whereas gradual slopes represent more open terrain, including relatively flat areas (Fig. A1.5).


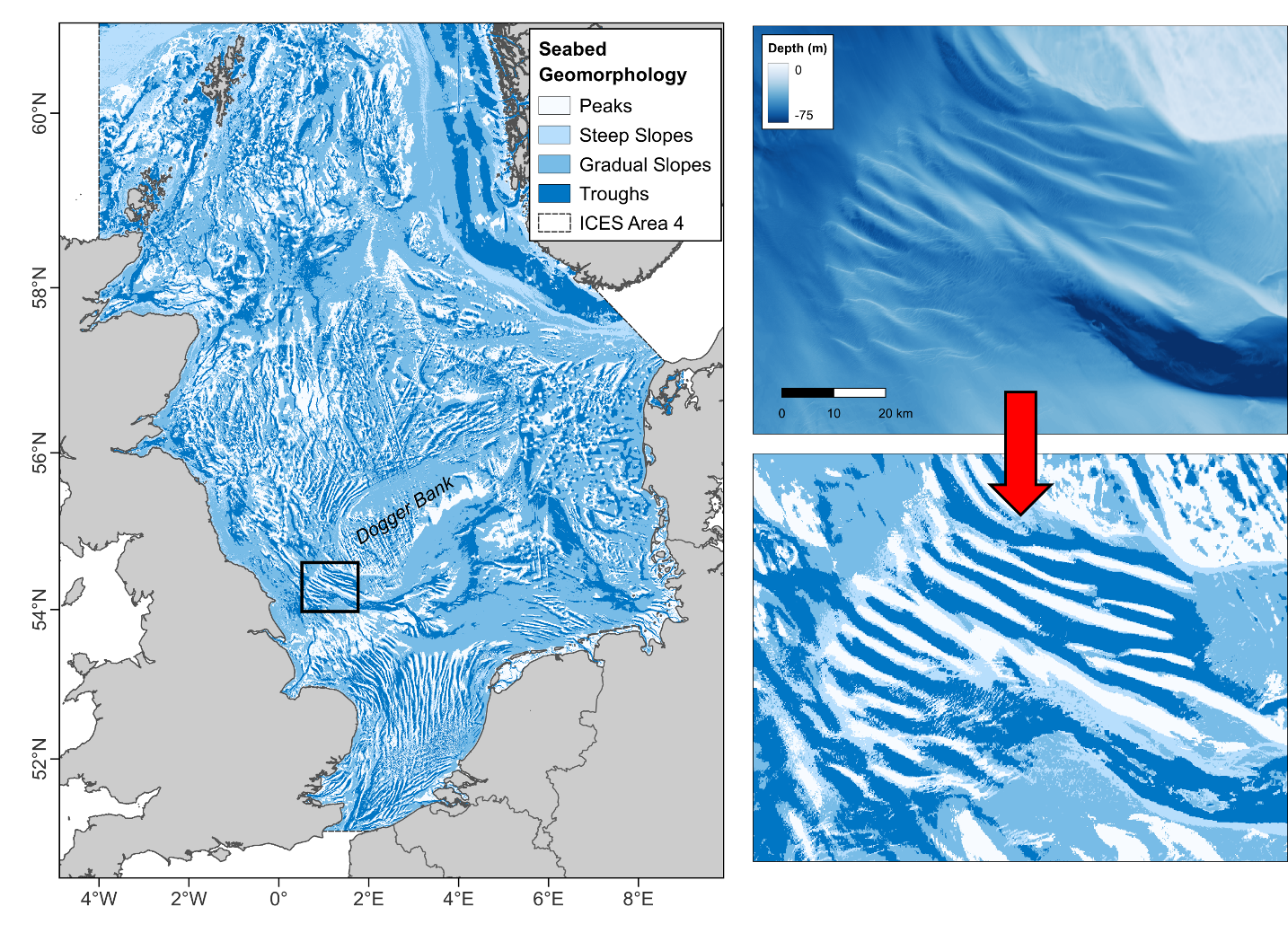


**Fig. A2**: Seabed geomorphology for the North Sea study area (ICES Area 4). Right hand graphics show bathymetric data for an area of sandbanks known as “The Hills” (marked with a black rectangle on the main map) southwest of the Dogger Bank, with the resulting geomorphology classifications shown below.

*Water Column Stratification*

For grey seals, vertical water column stratification during summer is an important predictor of habitat use in some regions (Carter et al., 2022). Summer mean potential energy anomaly (PEA) values were compiled from monthly PEA estimates for the European shelf (Jones, 2024) (Fig. A1.7). This covariate represents the amount of energy required in J/m^3^ to result in complete mixing of the water column under “typical” conditions for a given time of year. Thus, areas where the water column is fully mixed would have a PEA value of 0, and high values (> 100) are associated with areas of strong water column stratification. Values ~20 J/m^3^ roughly correspond to frontal regions where the transition from vertically mixed to stratified water occurs. This covariate was not included for harbour seals as there was little variation in PEA values experienced by the seals during the months coinciding with the considered tracking data.

*
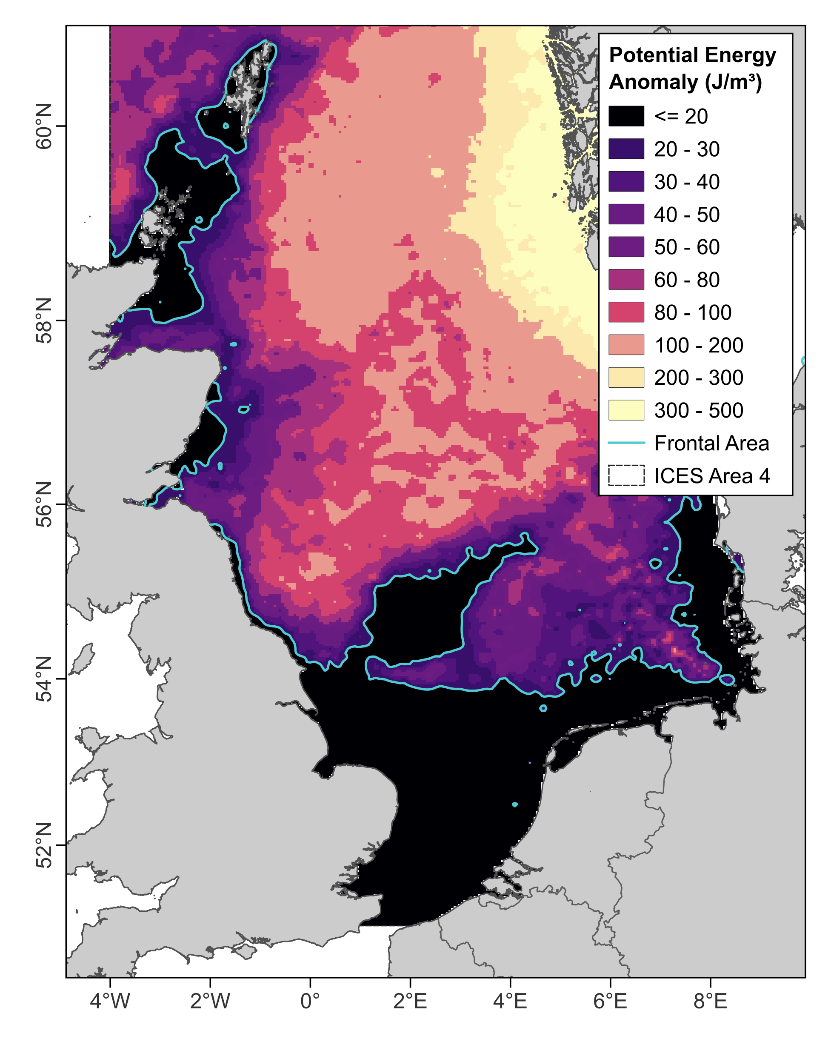
*

**Fig. A3**: Summer mean potential energy anomaly (PEA) for the North Sea study area (ICES Area 4). Values show the amount of energy required in J/m^3^ to result in complete mixing of the water column under typical conditions. Blue line shows the 20 J/m^3^ contour, which roughly correspond to frontal areas where vertically stratified and mixed waters meet. Data taken from Jones (2024).

1. **HMM Transition Probabilities**

**Table A1:** State transition probability matrix for transit and ARS states from HMMs fitted to grey and harbour seals. Values in parentheses show the 95% confidence intervals.

| **Grey**  **Seals** | | **To** | | |
| --- | --- | --- | --- | --- |
|  |  | **Transit** | **Broad ARS** | **Focussed ARS** |
| **From** | **Transit** | 0.85 (0.84-0.85) | 0.15 (0.14-0.15) | 0.004 (0.003-0.006) |
|  | **Broad ARS** | 0.08 (0.07-0.08) | 0.82 (0.82-0.83) | 0.10 (0.10-0.10) |
|  | **Focussed ARS** | 0.005 (0.004-0.007) | 0.16 (0.16-0.17) | 0.83 (0.83-0.84) |
|  | | | | |
| **Harbour**  **Seals** | | **To** | | |
|  |  | **Transit** | **Broad ARS** | **Focussed ARS** |
| **From** | **Transit** | 0.73 (0.73-0.74) | 0.25 (0.24-0.25) | 0.02 (0.02-0.03) |
|  | **Broad ARS** | 0.10 (0.09-0.10) | 0.78 (0.77-0.78) | 0.13 (0.13-0.13) |
|  | **Focussed ARS** | 0.02 (0.02-0.02) | 0.20 (0.20-0.21) | 0.78 (0.77-0.78) |

1. **Foraging Habitat Association Model Selection**

**Table A2: Backwards model selection by AIC score.** ∆AIC shows the change in AIC score in comparison to the top ranked model, Dev. shows the percentage deviance explained, “:” signifies an interaction between covariates. The full model for grey seals comprised a three-way interaction between geomorphology, substrate type and region, and an interaction between potential energy anomaly (PEA) and region. The full model for harbour seals comprised a three-way interaction between geomorphology, substrate type and region.

| **Rank** | **Covariates** | **ARS (Given Encounter)** | | | | **Focussed ARS (Given ARS)** | | | |
| --- | --- | --- | --- | --- | --- | --- | --- | --- | --- |
|  |  | **Grey** | | **Harbour** | | **Grey** | | **Harbour** | |
|  |  | **∆AIC** | **Dev.** | **∆AIC** | **Dev.** | **∆AIC** | **Dev.** | **∆AIC** | **Dev.** |
| 1 | Full model | - | 16.6 | - | 8.8 | - | 20.3 | - | 15.1 |
| 2 | Drop Substrate:Geomorphology:Region | 155.0 | 16.3 | 106.3 | 8.7 | 121.3 | 20.0 | 200.7 | 15.0 |
| 3 | Drop  PEA:Region | 744.5 | 15.6 | N/A | N/A | 192.5 | 20.0 | N/A | N/A |

1. **Harbour Seal Behaviour in The Dollart, Wadden Sea**

Of the tracked harbour seals hauling-out in the Wadden Sea region, 34 (20%) remained in The Dollart (an embayment in the inner Ems estuary) between offshore foraging trips (Fig. A4). Although the associated at-sea behaviour is largely classified as putative ARS by the HMM, given that seals also forage offshore, it is possible that this behaviour is more related to waiting for tidal haulouts to become available than to foraging. However, without simultaneously collected data (e.g., prey catch attempt metrics from accelerometers) to ground-truth the HMM state estimates, it is not possible to determine the true nature of this behaviour. Leaving this behaviour in the habitat association model may provide misleading results as these habitats are distinct from the other foraging areas in that they feature predominantly muddy substrate. The analysis presented in the main article therefore excludes locations in The Dollart, however here we present the results with those locations included. Retaining those locations shows a generally higher probability of ARS in muddy habitats than gravelly and sandy habitats (Fig. A5). Muddy habitats do not feature in the main results as they were not frequently encountered elsewhere in the region.


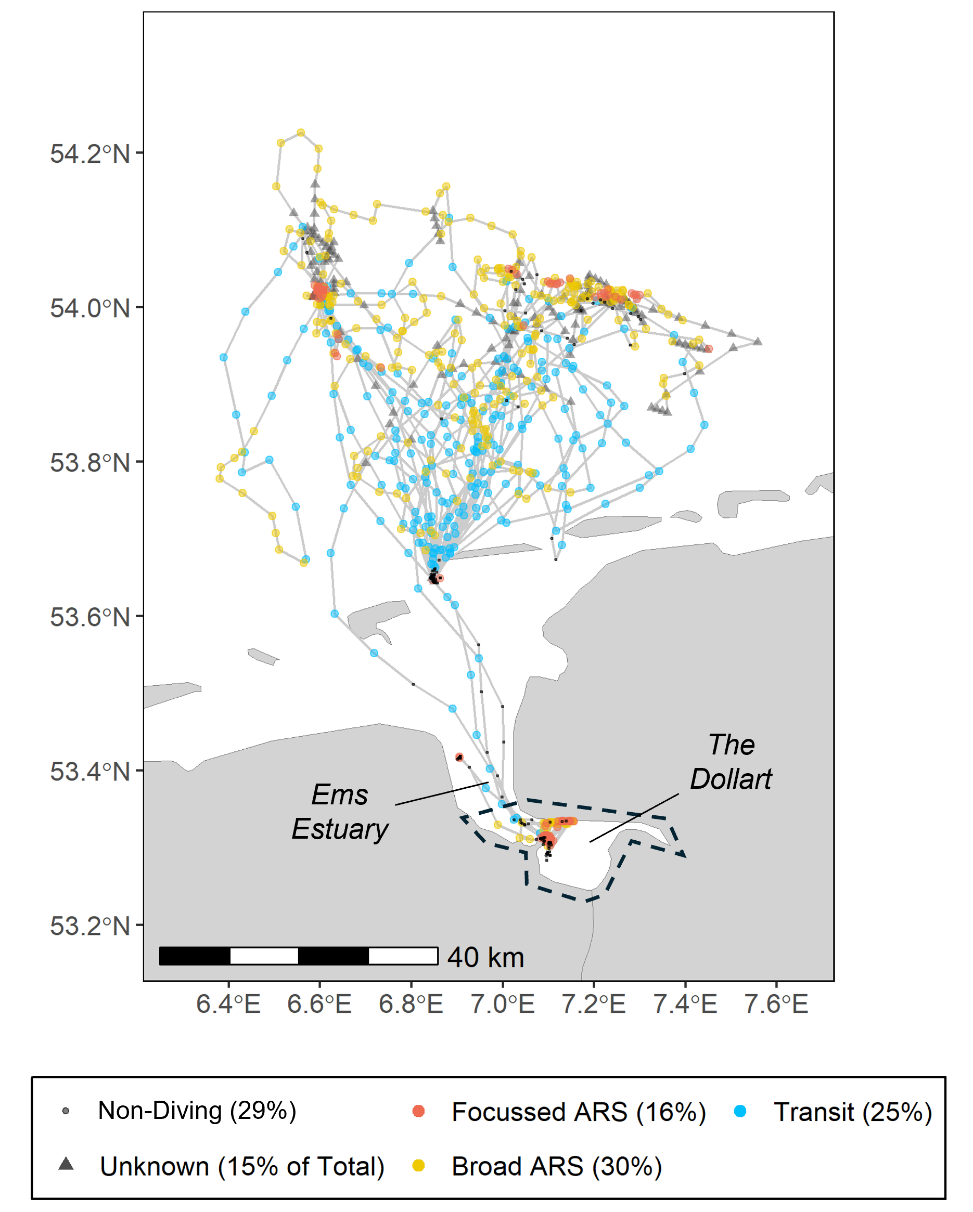


**Fig. A4:** Example track of a harbour seal tagged in the Wadden Sea region showing putative ARS behaviour within The Dollart. Coloured dots show the behavioural state assignments from the HMM. Surface locations indicate both haulout and at-sea surface behaviour. Dashed line indicates the area from which locations were removed in the main analysis.


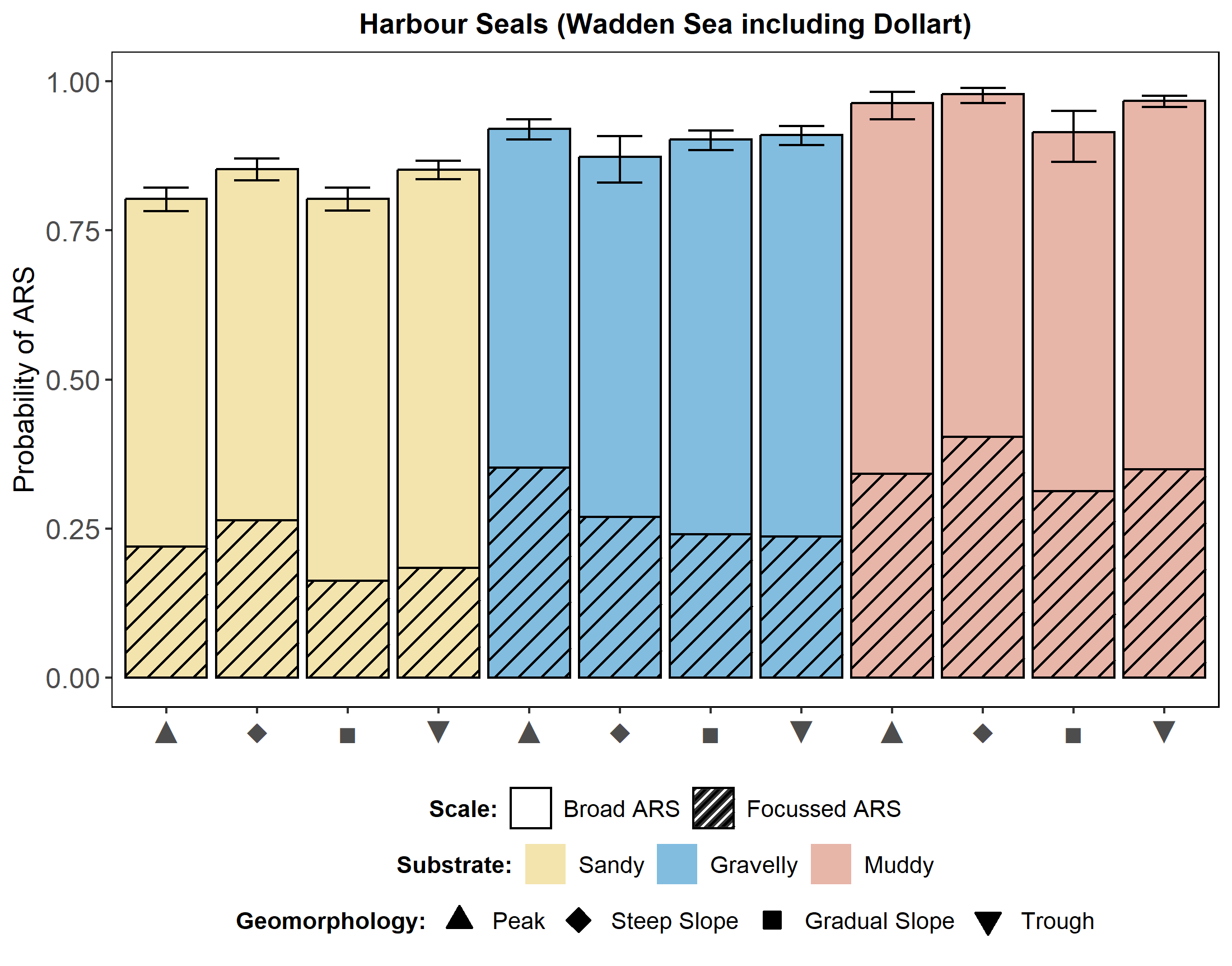


**Fig. A5:** Seabed foraging habitat associations of harbour seals hauling-out in the Wadden Sea with locations form The Dollart retained. Stacked bars show the mean probability of focussed ARS (hatched) and broad ARS (open) given encounter per habitat type. The sum of the two scales gives the overall probability of any ARS. The remaining probability space (up to 1) reflects the probability of being in transit state. Error bars show 95% confidence intervals around the mean probability of any ARS. Colours denote different substrate types, shapes denote different geomorphologies.

1. **Grey Seal Foraging Habitat Associations (Seabed Only)**

For comparability with harbour seals, the habitat association models for grey seals were fitted without the Potential Energy Anomaly (PEA) covariate (Fig. A6).


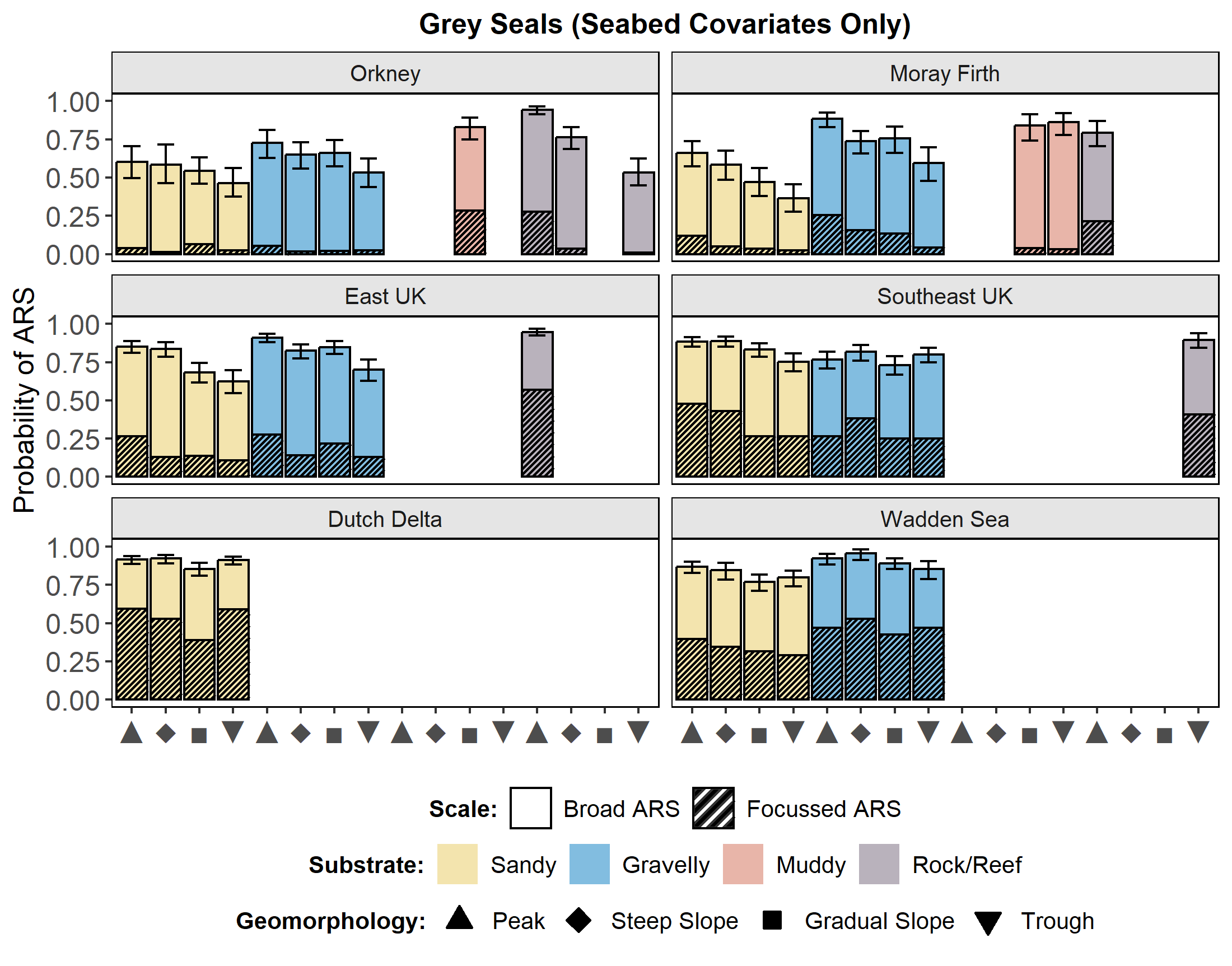


**Fig. A6:** Regional differences in seabed foraging habitat associations for grey seals. Stacked bars show the mean probability of focussed ARS (hatched) and broad ARS (open) given encounter per habitat type. The sum of the two scales gives the overall probability of any ARS. The remaining probability space (up to 1) reflects the probability of being in transit state. Error bars show 95% confidence intervals around the mean probability of any ARS. Colours denote different substrate types, shapes denote different geomorphologies. Habitat types that were rarely encountered (< 100 observations) are not shown.

**References**

Aarts, G., MacKenzie, M., McConnell, B., Fedak, M., & Matthiopoulos, J. (2008). Estimating space-use and habitat preference from wildlife telemetry data. *Ecography*, *31*, 140–160. https://doi.org/10.1111/j.2007.0906-7590.05236.x

Carter, M. I. D., Boehme, L., Cronin, M. A., Duck, C. D., Grecian, W. J., Hastie, G. D., Jessopp, M., Matthiopoulos, J., McConnell, B. J., Miller, D. L., Morris, C. D., Moss, S. E. W., Thompson, D., Thompson, P. M., & Russell, D. J. F. (2022). Sympatric seals, satellite tracking and protected areas: habitat-based distribution estimates for conservation and management. *Frontiers in Marine Science*, *9*, 875869. https://doi.org/10.3389/fmars.2022.875869

EMODnet Bathymetry. (2022). *EMODnet Digital Bathymetry (DTM 2022)*. https://emodnet.ec.europa.eu/en/bathymetry

EMODnet Seabed Habitats. (2021). *EMODnet Broad-scale Seabed Habitat Map for Europe (EUSeaMap)*. https://emodnet.ec.europa.eu/en/seabed-habitats

Jasiewicz, J., & Stepinski, T. F. (2013). Geomorphons - a pattern recognition approach to classification and mapping of landforms. *Geomorphology*, *182*, 147–156. https://doi.org/10.1016/j.geomorph.2012.11.005

Jones, S. (2024). *Monthly Potential Energy Anomaly (PEA) climatology for the European shelf [Data set]*. https://doi.org/10.5281/zenodo.10786742

Neteler, M., & Mitasova, H. (2007). *Open Source GIS: A GRASS GIS Approach* (3rd ed.). Springer Verlag.

Wyles, H. M. E., Boehme, L., Russell, D. J. F., & Carter, M. I. D. (2022). A novel approach to using seabed geomorphology as a predictor of habitat use in highly mobile marine predators: implications for ecology and conservation. *Frontiers in Marine Science*, *9*, 818635. https://doi.org/10.3389/fmars.2022.818635
